# Immune checkpoint inhibitors for extensive-stage small-cell lung cancer: a network meta-analysis and cost-effectiveness analysis

**DOI:** 10.3389/fimmu.2025.1662438

**Published:** 2025-10-27

**Authors:** Jiayu Wen, Yuhao Sun, Yuxuan Zhu, Jizhong Zhang, Qingshan Tang, Yifei Zhu, Nan Wu, Zhixian Liu, Xin Liu, Silu Xu, Jifu Wei, Guoren Zhou

**Affiliations:** ^1^ Jiangsu Cancer Hospital, Jiangsu Institute of Cancer Research, The Affiliated Cancer Hospital of Nanjing Medical University, Nanjing, Jiangsu, China; ^2^ School of Basic Medicine and Clinical Pharmacy, China Pharmaceutical University, Nanjing, China

**Keywords:** extensive-stage small-cell lung cancer, immune checkpoint inhibitors, cost-effectiveness analysis, network meta-analysis, partitioned survival model

## Abstract

**Background:**

Despite the established efficacy of immune checkpoint inhibitors (ICIs) in combination with chemotherapy, with or without anti-angiogenic agents, for extensive-stage small-cell lung cancer (ES-SCLC), a comprehensive comparative assessment of these regimens remains lacking. This study aimed to systematically compare the safety, efficacy, and cost-effectiveness of currently available ICI combination regimens for ES-SCLC.

**Methods:**

Phase III randomized clinical trials (RCTs) published up to January 31, 2025, were retrieved from PubMed, Web of Science, Cochrane Library, ClinicalTrials.gov, European Union Clinical Trials Register, and the Chinese Clinical Trial Registry. A network meta-analysis (NMA) was performed to evaluate overall survival (OS), progression-free survival (PFS), objective response rate (ORR), ≥grade 3 adverse events (AEs), and surface under the cumulative ranking curve analysis (SUCRA) score. A cost-effectiveness analysis (CEA) was performed from the perspective of the Chinese healthcare system. A 10-year partitioned survival model (PSM) was used to estimate total costs, quality-adjusted life-years (QALYs), and incremental cost-effectiveness ratios (ICERs).

**Results:**

Eight phase III RCTs were included. NMA demonstrated that benmelstobart and anlotinib plus chemotherapy ranked first in improving OS (HR = 0.81, 95% CI: 0.72–0.90), PFS (HR = 0.61, 95% CI: 0.55–0.67), and ORR (OR = 2.16, 95% CI: 1.43–3.27) compared to chemotherapy. Serplulimab plus chemotherapy ranked second in OS (HR = 0.82, 95% CI: 0.73–0.91) and PFS (HR = 0.73, 95% CI: 0.66–0.80). Regarding safety, tislelizumab plus chemotherapy exhibited the lowest incidence of ≥grade 3 AEs among eight ICI-based regimens. The CEA indicated that the ICERs of ICI-based regimens as compared to chemotherapy alone ranged from $45,360.61/QALY to $382,106.89/QALY, exceeding the Chinese willingness-to-pay (WTP) threshold ($40,500/QALY). However, tislelizumab plus chemotherapy emerged as relatively cost-effective, achieving the second-highest QALYs (1.27) and the second-lowest costs ($52,273.46). Sensitivity analyses affirmed the robustness of these findings.

**Conclusions:**

Benmelstobart and anlotinib plus chemotherapy demonstrated superior efficacy regarding OS, PFS, and ORR, while tislelizumab plus chemotherapy provided the most favorable safety profile among the evaluated ICI-based therapies. And none of the evaluated ICI-based regimens were cost-effective at the conventional WTP threshold. However, tislelizumab plus chemotherapy was the most cost-effective as the WTP threshold increased.

## 1 Introduction

Lung cancer is a growing human health concern and ranked first in incidence (22.0%) and mortality (28.5%) in China (1). According to the 2022 global cancer statistics, there were approximately 2.5 million new cases and 1.8 million cancer-related deaths worldwide in 2022 (2). Lung cancer comprises two main subtypes: non-small cell lung cancer (NSCLC) and small cell lung cancer (SCLC). SCLC accounts for around 15% of newly diagnosed lung cancers (3). Due to insidious symptoms and rapid metastasis, over half of the SCLC patients are diagnosed at an advanced stage (4). Extensive-stage SCLC (ES-SCLC) has drawn significant attention due to its rapid proliferation, predisposing to distant metastases and drug resistance (5).

Historically, platinum-based chemotherapy has served as the standard first-line treatment for ES-SCLC. However, platinum-based chemotherapy provides limited clinical benefits, which are reflected by a median overall survival (OS) of approximately 10 months and a two-year survival rate of less than 5% (6, 7). The emergence of immunotherapy, particularly immune checkpoint inhibitors (ICIs) targeting programmed cell death protein 1 (PD-1), programmed cell death-ligand 1 (PD-L1), and cytotoxic T-lymphocyte-associated protein 4 (CTLA-4), has revolutionized the therapeutic landscape of ES-SCLC, improving the OS and progression-free survival (PFS) of patients (8). Numerous phase III clinical trials have confirmed that the combination of ICIs and platinum-based chemotherapy can significantly improve clinical outcomes as compared to chemotherapy alone in ES-SCLC patients (9–16). Despite this progress, there is limited improvement in the long-term survival for ES-SCLC patients, with a median OS gain of approximately 3 months, thus highlighting an ongoing unmet clinical need (9–14, 16). Recent studies have highlighted the complexity of the ES-SCLC tumor microenvironment, which is characterized by immunosuppression and angiogenesis. The ETER701 trial reported that the combination of chemo-immunotherapy with anti-angiogenic agents, such as anlotinib, could significantly extend the median OS from 11.9 to 19.3 months (approximately 7.4 months) compared to chemotherapy alone (15, 17). The combination of anti-angiogenic agents with ICIs has shown synergistic effects, suggesting improvements in angiogenesis regulation, immune microenvironment enhancement, and potential reversal of drug resistance (18, 19).

While combination therapies promise extended survival benefits, they also significantly increase the financial burden on the patients due to their high costs. Therefore, evaluating the relative cost-effectiveness of various treatment regimens is critical. Currently, the studies investigating comprehensive comparisons among emerging ICI-based regimens, including novel combinations, such as benmelstobart combined with anlotinib and chemotherapy, are lacking. Consequently, an optimal combination therapy for achieving maximum long-term survival benefits and cost-effectiveness remains unclear, further complicating clinical decision-making. In order to address this knowledge gap, a comprehensive network meta-analysis (NMA) and cost-effectiveness analysis (CEA) from the perspective of the Chinese healthcare system were conducted. This study aimed to evaluate and compare the efficacy, safety, and cost-effectiveness of various ICI-based combination therapies, thus providing valuable guidance to clinicians and promoting rational medication use in clinical practice.

## 2 Methods

### 2.1 Network meta-analysis

This study was performed in accordance with the Preferred Reporting Items for Systematic Reviews and Meta-Analyses (PRISMA) statement, including the PRISMA NMA checklist (**Supplementary Table S1**). The data used in this study are publicly accessible, without direct intervention or individual patient-level data collection; thus, institutional ethics approval was waived.

#### 2.1.1 Study selection and inclusion criteria

PubMed, Web of Science, Cochrane Library, ClinicalTrials.gov, European Union Clinical Trials Register, and the Chinese Clinical Trial Registry were systematically searched up to January 31, 2025. The search terms included “extensive-stage small cell lung cancer”, “durvalumab”, “atezolizumab”, “serplulimab”, “pembrolizumab”, “tislelizumab”, “benmelstobart”, “toripalimab”, “adebrelimab”, and “clinical trials” (**Supplementary Table S2**). These eight ICIs were selected because they represent all ICIs with available phase III randomized trial data in ES-SCLC using platinum–based chemotherapy as the control arm. Moreover, they are all listed as recommended treatment options in the Chinese Society of Clinical Oncology (CSCO) 2025 guidelines for ES-SCLC, thereby covering the regimens most widely supported by phase III evidence and current guidelines in this setting (20). Two researchers independently screened articles according to the PICOS (Patients, Intervention, Comparison, Outcome, and Study design) criteria to ensure comprehensive and high-quality data collection. Any disagreement between the researchers was addressed by consulting with GRZ researcher.

Eligible studies met the following inclusion criteria: (1) the enrolled patients were diagnosed with ES-SCLC; (2) treatment regimens included ICIs combined with etoposide-platinum-based chemotherapy, with or without an anti-angiogenic agent; (3) phase III randomized controlled trials (RCTs); (4) the studies reporting comprehensive, up-to-date efficacy and safety outcomes, including OS, PFS, objective response rate (ORR), and incidence of ≥grade 3 adverse events (AEs); (5) drug prices available in the local database (https://www.pharnexcloud.com/). Any article that failed to meet the inclusion criteria was excluded.

#### 2.1.2 Data extraction and quality assessment

The data extracted from the eligible studies included trial characteristics (sample size, intervention arm, and control arm) and clinical outcomes (ORR, OS, PFS, and ≥ grade 3 AEs) (**Supplementary Table S3**). The bias risk was assessed according to the Cochrane Collaboration guidelines using Review Manager 5.3, evaluating random sequence generation, allocation concealment, blinding of participants and personnel, blinding of outcome assessment, incomplete outcome data, selective reporting, and other potential biases (21).

#### 2.1.3 Statistical analyses

Heterogeneity was assessed using Review Manager 5.3 software. Based on the *I*² test results, the fixed-effect model was used to analyze the OS and ≥grade 3 AEs, while the random-effect model was used to analyze the PFS and ORR. Hazard ratios (HRs) with 95% confidence intervals (CIs) were calculated for the OS and PFS, and risk ratios (RRs) with 95% CIs were computed for ≥grade 3 AEs and ORR using the R software package “netmeta” (version 4.4.1, https://www.r-project.org/). Indirect comparative meta-analyses were further performed using the R software packages “Gemtc” and “ADDIS”. The efficacy and safety profiles of the evaluated regimens were ranked based on OS, PFS, ORR, and ≥grade 3 AEs using the surface under cumulative ranking analysis (SUCRA). No pooled pseudo–individual-level dataset was created; all NMA comparisons were based on study-level aggregate outcomes, ensuring that each trial was analyzed independently.

### 2.2 Cost-effectiveness analysis

This study was conducted in accordance with the Consolidated Health Economic Evaluation Reporting Standards (CHEERS) checklist (22). The detailed data are provided in **Supplementary Table S4**. The economic analysis was performed from the perspective of China’s healthcare system.

#### 2.2.1 Patients and therapeutic regimens

The control group regimen included chemotherapy with the intravenous administration of carboplatin (AUC = 5, day 1) and etoposide (100 mg/m²/day, days 1–3), which was repeated every 3 weeks for four cycles, followed by placebo maintenance. The experimental group regimen included this chemotherapy in combination with ICIs (administered intravenously on day 1 of each cycle) for four cycles. The ICIs included adebrelimab (20 mg/kg), pembrolizumab (200 mg), serplulimab (4.5 mg/kg), tislelizumab (200 mg), atezolizumab (1200 mg), durvalumab (1500 mg), benmelstobart (1200 mg), and toripalimab (240 mg). In the benmelstobart arm, oral anlotinib (12 mg daily on days 1–14 of each cycle) was added. The subsequent maintenance involved either ICIs alone or in combination with anlotinib and terminated immediately upon progressive disease (PD). Given the absence of second-line treatment details in the included trials, subsequent therapy upon PD was standardized to topotecan monotherapy (days 1–5 every 3 weeks); this was done in accordance with the 2025 Chinese Society of Clinical Oncology (CSCO) SCLC guidelines (20).

#### 2.2.2 Model construction

**Figure 1:**
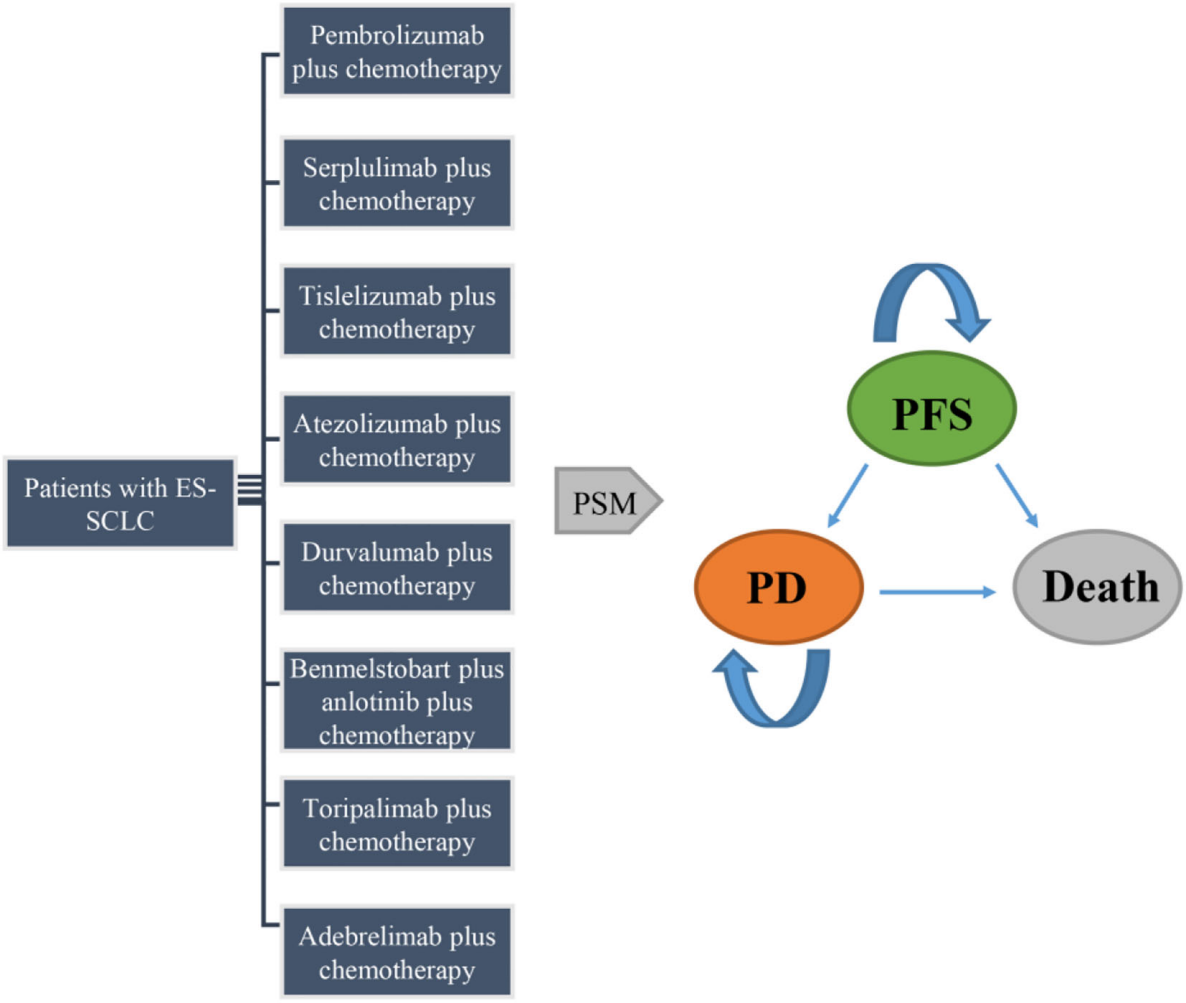
Structure of the partitioned survival model. ES-SCLC, extensive-stage small-cell lung cancer; PFS, progression-free survival; PD, progressed disease; PSM, partitioned survival model.

A partitioned survival model (PSM) with three health states (PFS, PD, and death) was established to evaluate the cost-effectiveness (**Figure 1**). The model adopted a 3-week cycle duration and a 10-year horizon. Initially, all the patients were in the PFS state, transitioning to PD and death states over time. The costs and utilities were discounted at 5% annually according to the China Guidelines for Pharmacoeconomic Evaluations (2020) (23). The outcomes included total costs, quality-adjusted life-years (QALYs), and incremental cost-effectiveness ratios (ICERs). ICERs were calculated as the primary evaluation indicator, representing the incremental cost per additional QALY. All ICERs were calculated separately within each trial by comparing each ICI regimen with its corresponding chemotherapy control, ensuring that the analyses were based solely on study-level data. Willingness-to-pay (WTP) was set at $40,500 per QALY, which is three times China’s 2024 GDP per capita and aligns with World Health Organization (WHO) guidelines (24, 25). A regimen whose ICER falls below the predetermined WTP threshold was considered cost-effective, while one exceeding the threshold was not.

#### 2.2.3 Cost inputs and utility estimates

Data were extracted from the OS and PFS curves of RCTs using the GetData Graph Digitizer (version 2.26; https://getdata-graph-digitizer.com). The extracted data were subsequently refined to ensure that survival rates remained stable or declined over time, thereby eliminating any implausible upward trends. The processed data were then arranged into two datasets: one containing the number of risk events over time, and the other containing survival rates corresponding to specific time points for OS or PFS. Individual patient data were reconstructed using the R package ‘digitise,’ following an approach consistent with the established Guyot method for survival data reconstruction. Based on the reconstructed individual patient data, Kaplan–Meier curves were generated using the R package ‘survminer’ (**Supplementary Figure S1** and **S2**). Subsequently, parametric survival models were developed and evaluated to project long-term outcomes, allowing extrapolation of survival data over a 10-year horizon for the cost-effectiveness analysis. In parametric model construction, log-logistic distribution was selected as the best-fitting parameter model based on Akaike information criterion (AIC) and Bayesian information criterion (BIC) (**Supplementary Figures S3**, **S4**; **Supplementary Table S5**) (26). The log-logistic distribution was selected as the preferred model for survival extrapolation, as it achieved the lowest AIC and BIC values in most trials. It provided the best fit for OS in CAPSTONE-1, ASTRUM-005, RATIONALE-312, and EXTENTORCH, while in KEYNOTE-604 and ETER701 some survival curves also favored the log-logistic distribution. In IMpower133 and CASPIAN, although alternative models produced slightly lower AIC/BIC values, the differences from the log-logistic model were marginal (≤19), indicating no statistically meaningful superiority of alternative models. Therefore, the log-logistic distribution was applied as a unified model to ensure methodological consistency and comparability across analyses. The shape (γ) and scale (λ) parameters of the log-logistic distribution were estimated using the R software (**Supplementary Table S6**). Health state indices were derived from the EQ-5D-5L user guide, where 0.70, 0.6, and 0 indicated PFS, PD, and death, respectively (27). This cost-effectiveness analysis was conducted from the perspective of the Chinese healthcare system, and therefore only direct medical costs were considered, including drug acquisition, AE management, follow-up testing, subsequent therapy, and best supportive care (BSC). The direct medical costs of drug acquisition were acquired from the local database (https://www.pharnexcloud.com/). Drug administration schedules in the cost-effectiveness analysis were aligned with those reported in the clinical trials (9–16). For agents with dosing based on body weight or body surface area, we assumed a representative patient with an average body weight of 64 kg, a body surface area of 1.72 m² (28) and a creatinine clearance of 80 mL/min (29). For AE management, follow‐up testing, subsequent therapy, and BSC, cost inputs were obtained from literature data (30, 31). The related cost breakdowns and their references are provided in **Supplementary Table S7**. All costs were converted to US dollars (1 USD = 7.23 RMB, March 2025).

#### 2.2.4 Sensitivity analyses

One-way analysis and probabilistic sensitivity analysis (PSA) were performed using the R software to assess uncertainty. The one-way analysis evaluated changes in parameters (± 20% for cost inputs and utilities, ± 40% for discount rate), which were then illustrated using tornado diagrams. PSA was performed using a Monte-Carlo simulation (1,000 iterations), and the results were presented using cost-effectiveness scatter plots and acceptability curves. One-way sensitivity analyses and scatter plots were likewise conducted separately within each trial. For the CEAC, however, study-level control arm data were averaged across all trials to provide a common chemotherapy reference against which each experimental regimen was compared.

## 3 Results

### 3.1 Network meta-analysis

#### 3.1.1 Literature review and quality assessment

A total of 1,041 articles were initially identified from the databases. After rigorous screening, eight phase III RCTs, including CAPSTONE-1, KEYNOTE-604, ASTRUM-005, RATIONALE-312, IMpower133, CASPIAN, ETER701, and EXTENTORCH (9–16, 32, 33) were used in the NMA (**Supplementary Figure S5**). The NMA network diagram is shown in **Supplementary Figure S6**. Across the eight included RCTs, a total of 1820 patients were randomized to chemotherapy-alone control arms, while the experimental arms included chemotherapy combined with ICIs, with or without an anti-angiogenic agent: adebrelimab (n = 230), pembrolizumab (n = 228), serplulimab (n = 389), tislelizumab (n = 227), atezolizumab (n = 201), durvalumab (n = 268), benmelstobart plus anlotinib (n = 246), and toripalimab (n = 223).

The risk of bias assessment indicated that most trials had low bias risk, except the CASPIAN study, which was an open-label, sponsor-blind clinical trial, and the IMpower133 study, which lacked blinding in outcome assessment (**Supplementary Figure S7**). Heterogeneity was evaluated using the *I*² statistics, indicating fixed-effect models for OS (*I*² = 0%) and ≥grade 3 AEs (*I*² = 0%) and random-effect models for PFS (*I*² >50%) and ORR (*I*² = 43%).

#### 3.1.2 Efficacy

**Figure 2:**
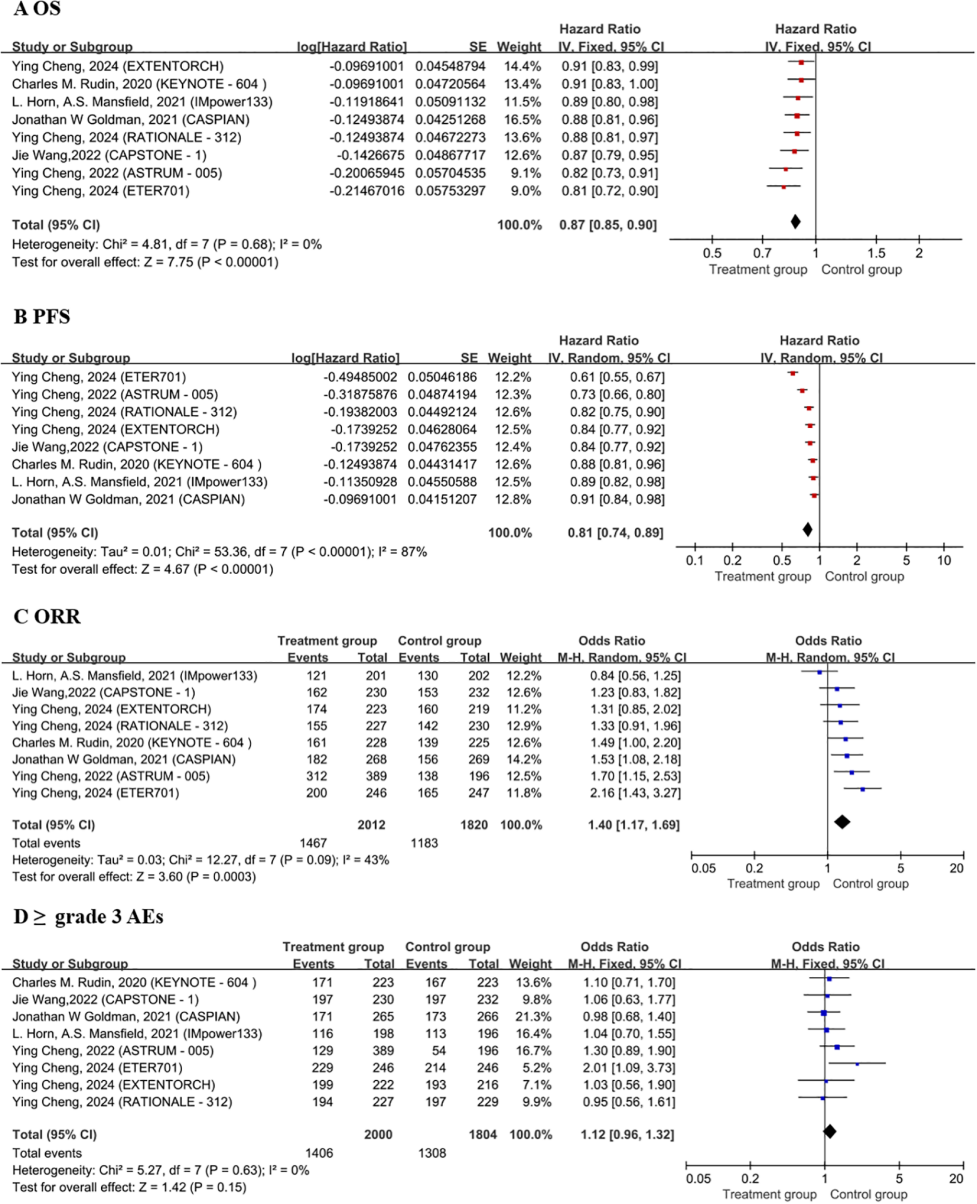
Forest plots of OS **(A)**, PFS **(B)**, ORR **(C)**, ≥ grade 3 AEs **(D)**. OS, overall survival; PFS, progression-free survival; ORR, objective response rate; AEs, adverse events; CT, Chemotherapy.

**Figure 3:**
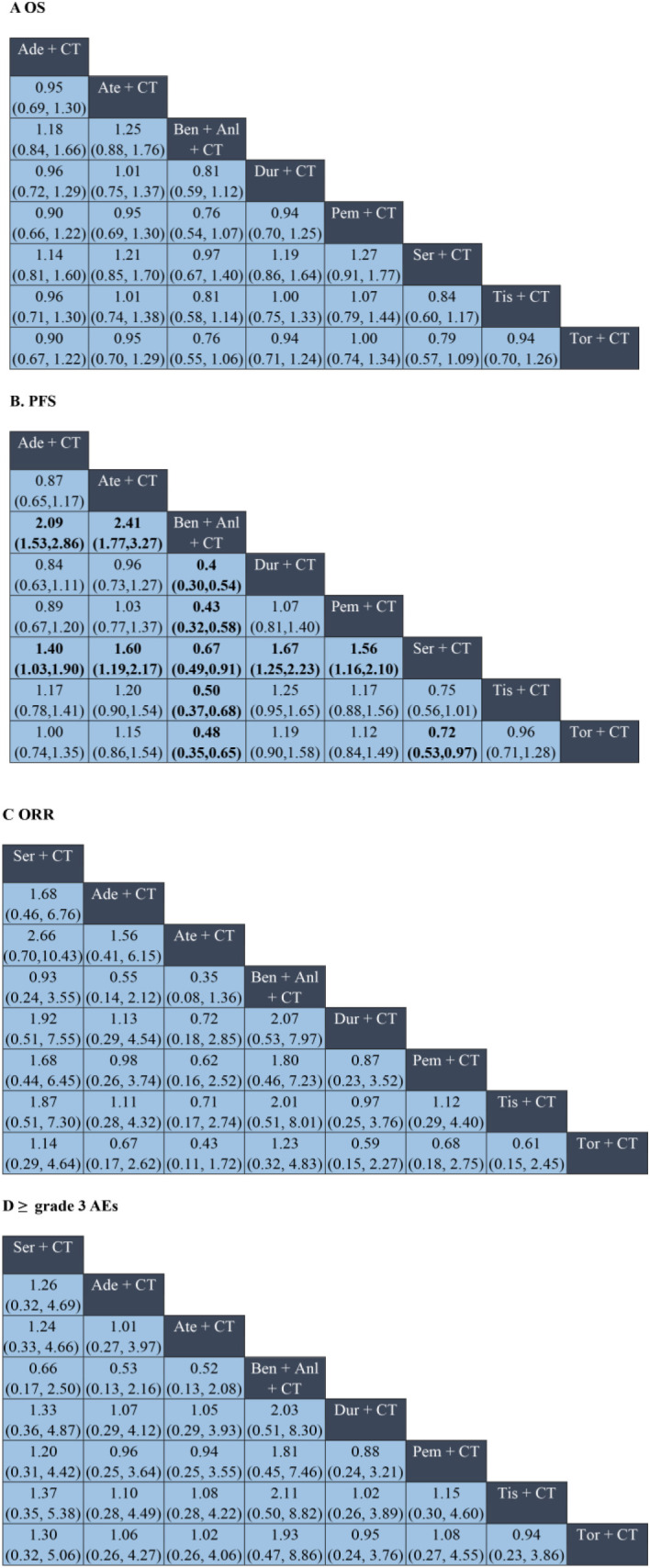
Network meta-analysis of ICI plus chemotherapy and ICI plus chemotherapy and anti-angiogenic regimens in ES-SCLC. Panels: **(A)** overall survival (OS); **(B)** progression-free survival (PFS); **(C)** objective response rate (ORR); **(D)** ≥grade 3 adverse events (AEs). In **(A, B)**, cells display hazard ratios (HRs); in **(C, D)**, cells display odds ratios (ORs). Cells are formatted as “effect estimate (95% CI)”; HR <1 (OR >1) indicates a superior row regimen than the column regimen; confidence intervals not crossing 1 denote statistical significance. Ade, adebrelimab; Ate, atezolizumab; Ben, benmelstobart; Anl, anlotinib; Dur, durvalumab; Pem, pembrolizumab; Ser, serplulimab; Tis, tislelizumab; Tor, toripalimab; CT, chemotherapy; CI, confidence interval.

The OS analysis included eight treatment strategies from eight studies. In terms of the HRs for OS, as compared to chemotherapy alone, the combination of chemotherapy with benmelstobart plus anlotinib significantly reduced mortality risk (HR = 0.81, 95% CI: 0.72–0.90), followed closely by combination of chemotherapy with serplulimab (HR = 0.82, 95% CI: 0.73–0.91) (**Figure 2A**). Although the combination of chemotherapy with benmelstobart plus anlotinib showed a trend towards delayed disease progression (all HRs <1), no superiority was observed against other ICI combination therapies (**Figure 3A**). For PFS, all eight combination therapies significantly outperformed chemotherapy alone, with the combination of chemotherapy with benmelstobart plus anlotinib exhibiting the most substantial benefit (HR = 0.61, 95% CI: 0.55–0.67), followed by combination of chemotherapy with serplulimab (HR = 0.73, 95% CI: 0.66–0.80) (**Figure 2B**). Furthermore, benmelstobart exhibited the greatest clinical benefit compared to other ICI combination therapy regimens (**Figure 3B**).

Regarding ORR, the combination of chemotherapy with durvalumab (OR = 1.53, 95% CI: 1.08–2.18), serplulimab (OR = 1.70, 95% CI: 1.15–2.53), and benmelstobart (OR = 2.16, 95% CI: 1.43–3.27) showed significant improvements as compared to chemotherapy alone (**Figure 2C**). However, no significant differences were observed among ICIs regimens in the indirect comparisons. The details are presented in **Figure 3C**.

#### 3.1.3 Safety

Safety analysis focused on the ≥grade 3 AEs according to the Common Terminology Criteria for Adverse Events (CTCAE). Among all the combination therapies, the combination of chemotherapy with benmelstobart plus anlotinib showed significantly higher ≥grade 3 AEs incidence as compared to chemotherapy alone (OR = 2.01; 95% CI: 1.09-3.73) (**Figure 2D**). No significant differences were noted between ICI-based regimens (**Figure 3D**).

#### 3.1.4 Treatment ranking

Treatment ranking probabilities were estimated using the SUCRA values across eight ICI-based regimens. For OS and PFS, the combination of chemotherapy with benmelstobart and anlotinib demonstrated the highest ranking (SUCRA 95.4% and 99.9%), followed by the combination of chemotherapy with serplulimab (SUCRA 91.0% and 86.8%). In terms of ORR, the combination of chemotherapy with benmelstobart and anlotinib also ranked first (SUCRA 87.5%), followed by the combination of chemotherapy with durvalumab (SUCRA 74.4%). Regarding safety, the tislelizumab- and durvalumab-based regimens exhibited the most favorable profiles, with SUCRA values of 67.1% and 65.0% for the lowest incidence of ≥grade 3 AEs, while the benmelstobart- and serplulimab-based regimens showed higher toxicity. The detailed results are listed in **Table 1**.

**Table 1:** SUCRA sortings of clinical efficacy and safety for different treatment options.

Group	OS		PFS		ORR		≥grade 3 AEs	
	SUCRAs	Rank	SUCRAs	Rank	SUCRAs	Rank	SUCRAs	Rank
Ben + anl + CT	0.954	1	0.999	1	0.875	1	0.216	7
Tor + CT	0.262	8	0.549	4	0.407	7	0.610	3
Dur + CT	0.506	4	0.248	8	0.744	2	0.650	2
Pem + CT	0.264	7	0.354	6	0.661	3	0.486	6
Ser + CT	0.910	2	0.868	2	0.655	4	0.120	8
Tis + CT	0.504	5	0.622	3	0.540	5	0.671	1
Ade + CT	0.647	3	0.548	5	0.410	6	0.572	4
Ate + CT	0.452	6	0.311	7	0.060	8	0.532	5

CT, Chemotherapy; Ate, Atezolizumab; Dur, Durvalumab; Ser, Serplulimab; Ade, Adebrelimab; Pem, Pembrolizumab; Ben, Benmelstobart; Tis, Tislelizumab; Tor, Toripalimab; anl, anlotinib; OS, overall survival; PFS, progression-free survival; ORR, objective response rate; AEs, adverse events; SUCRA, surface under cumulative ranking analysis.

### 3.2 Cost-effectiveness analysis

#### 3.2.1 Base-case analysis

A partitioned survival model with a 10-year time horizon was established. As listed in **Table 2**, the ICERs ranged from $45,360.61/QALY to $382,106.89/QALY, exceeding the WTP threshold of $40,500/QALY. This indicated that none of the ICI‐based regimens was cost-effective as compared to chemotherapy alone. A stepwise, incremental analysis of the ICERs across the eight ICI-based combination regimens was performed. The results showed that tislelizumab exhibited favorable cost-effectiveness with the second-highest QALYs (1.27) and second-lowest costs ($52,273.46), whereas durvalumab was the least cost-effective (lowest QALYs at 1.03 and highest costs at $132,837.11).

**Table 2:** Baseline cost-effectiveness analysis results over a 10-year horizon.

Strategy		Costs	Incremental costs	Overall QALYs	Incremental QALYs	ICER
CAPSTONE-1	Adebrelimab + Chemotherapy	81043.68	48722.96	1.13	0.30	162409.87
	Chemotherapy	32320.72	NA	0.83	NA	NA
KEYNOTE-604	Pembrolizumab + Chemotherapy	83439.54	55800.26	0.94	0.24	232501.08
	Chemotherapy	27639.28	NA	0.70	NA	NA
ASTRUM-005	Serplulimab + Chemotherapy	72631.85	39736.47	1.19	0.36	110379.08
	Chemotherapy	32895.38	NA	0.83	NA	NA
RATIONALE-312	Tislelizumab + Chemotherapy	52273.46	17237.03	1.27	0.38	45360.61
	Chemotherapy	35036.43	NA	0.89	NA	NA
IMpower133	Atezolizumab + Chemotherapy	86022.61	54682.52	1.09	0.29	188560.41
	Chemotherapy	31340.09	NA	0.80	NA	NA
CASPIAN	Durvalumab + Chemotherapy	132837.11	103168.86	1.03	0.27	382106.89
	Chemotherapy	29668.25	NA	0.76	NA	NA
ETER701	Benmelstobart + Anlotinib + Chemotherapy	102934.50	70031.04	1.31	0.48	145898.00
	Chemotherapy	32903.46	NA	0.83	NA	NA
EXTENTORCH	Toripalimab + Chemotherapy	42931.62	8895.87	1.05	0.18	49421.50
	Chemotherapy	34035.75	NA	0.87	NA	NA

#### 3.2.2 Sensitivity analysis

**Figure 4:**
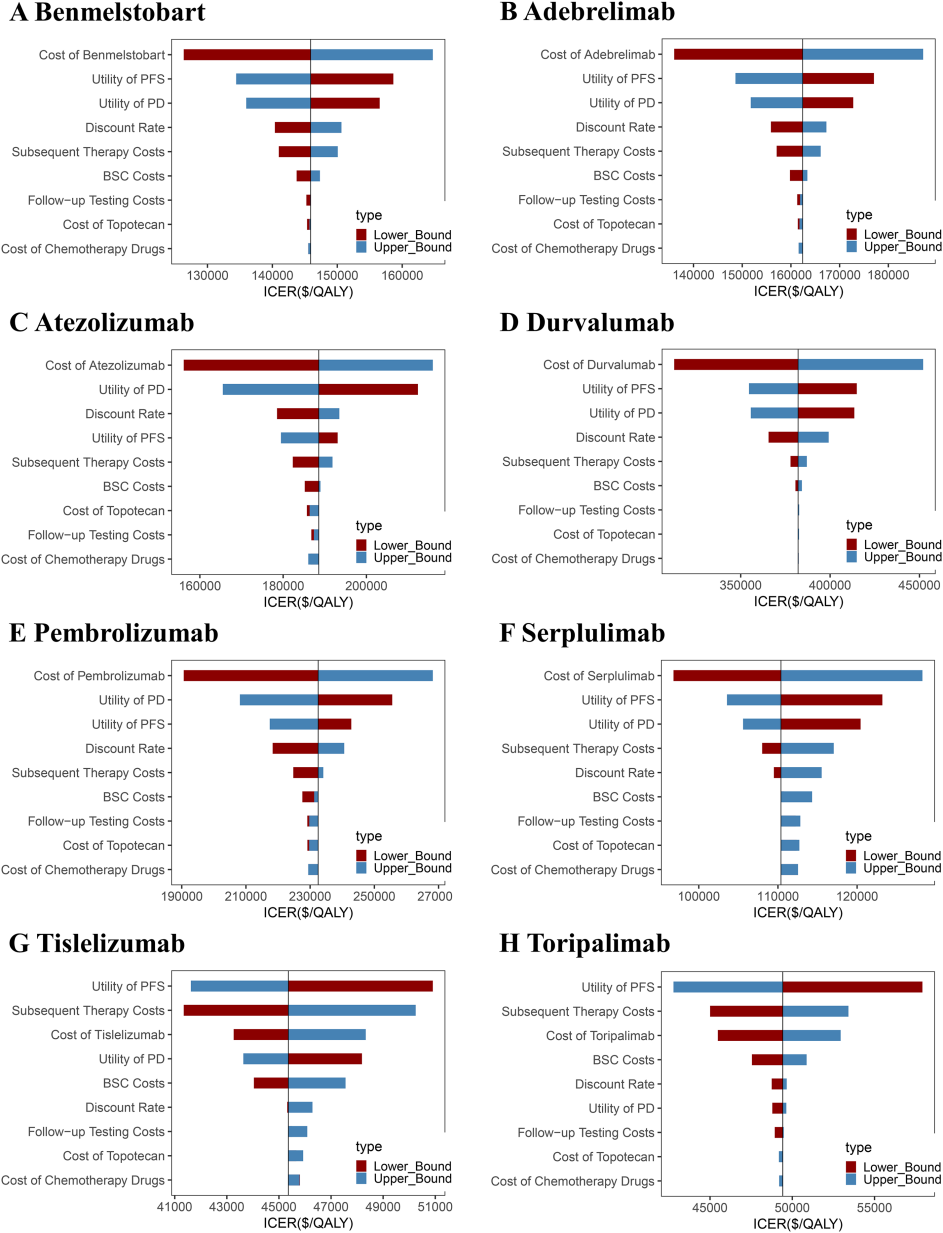
One-way sensitivity analysis tornado diagrams showing the parameters with the greatest impact on the ICER of each ICI-based regimen as compared to chemotherapy alone. **(A)** Benmelstobart, **(B)** Adebrelimab, **(C)** Atezolizumab, **(D)** Durvalumab, **(E)** Pembrolizumab, **(F)** Serplulimab, **(G)** Tislelizumab, and **(H)** Toripalimab. PFS, progression-free survival; PD, progressive disease; ICER, incremental cost-effectiveness ratio; BSC, best supportive care.

One-way sensitivity analysis revealed that, as compared to chemotherapy alone, the ICER values for adebrelimab, benmelstobart, durvalumab, pembrolizumab, and serplulimab regimens were most sensitive to variations in drug costs, PFS, and PD. Drug costs, PD, and discount rate showed the largest impact on the ICER of atezolizumab. ICER values for tislelizumab and toripalimab were the most sensitive to PFS, subsequent therapy costs, and drug costs. Nevertheless, even under optimal parameter variations, none of the treatments met the cost-effectiveness threshold (**Figure 4**).

**Figure 5:**
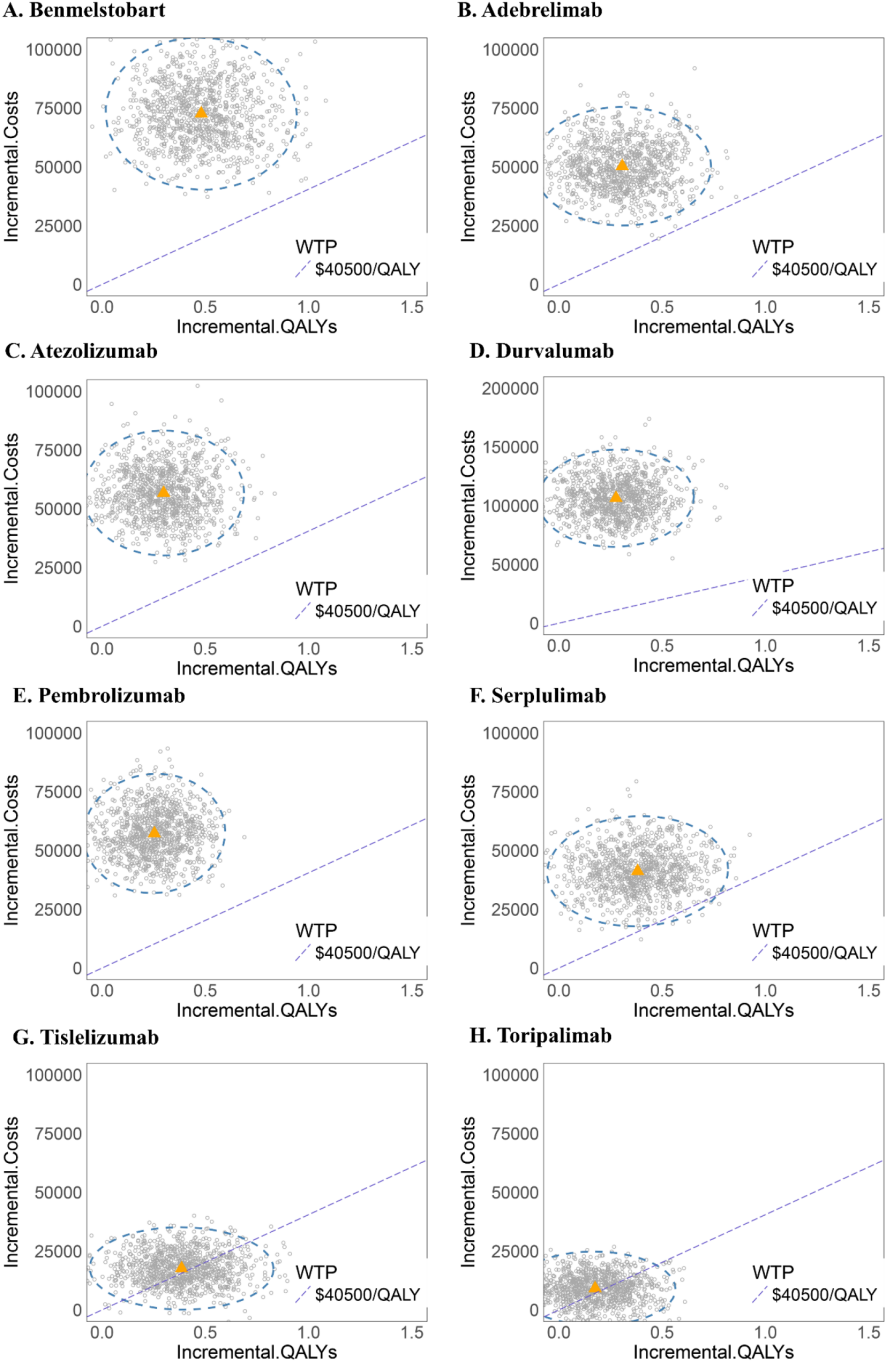
Probabilistic sensitivity analysis scatter plots for each ICI-based regimen as compared to chemotherapy alone. **(A)** Benmelstobart, **(B)** Adebrelimab, **(C)** Atezolizumab, **(D)** Durvalumab, **(E)** Pembrolizumab, **(F)** Serplulimab, **(G)** Tislelizumab, and **(H)** Toripalimab.

**Figure 6:**
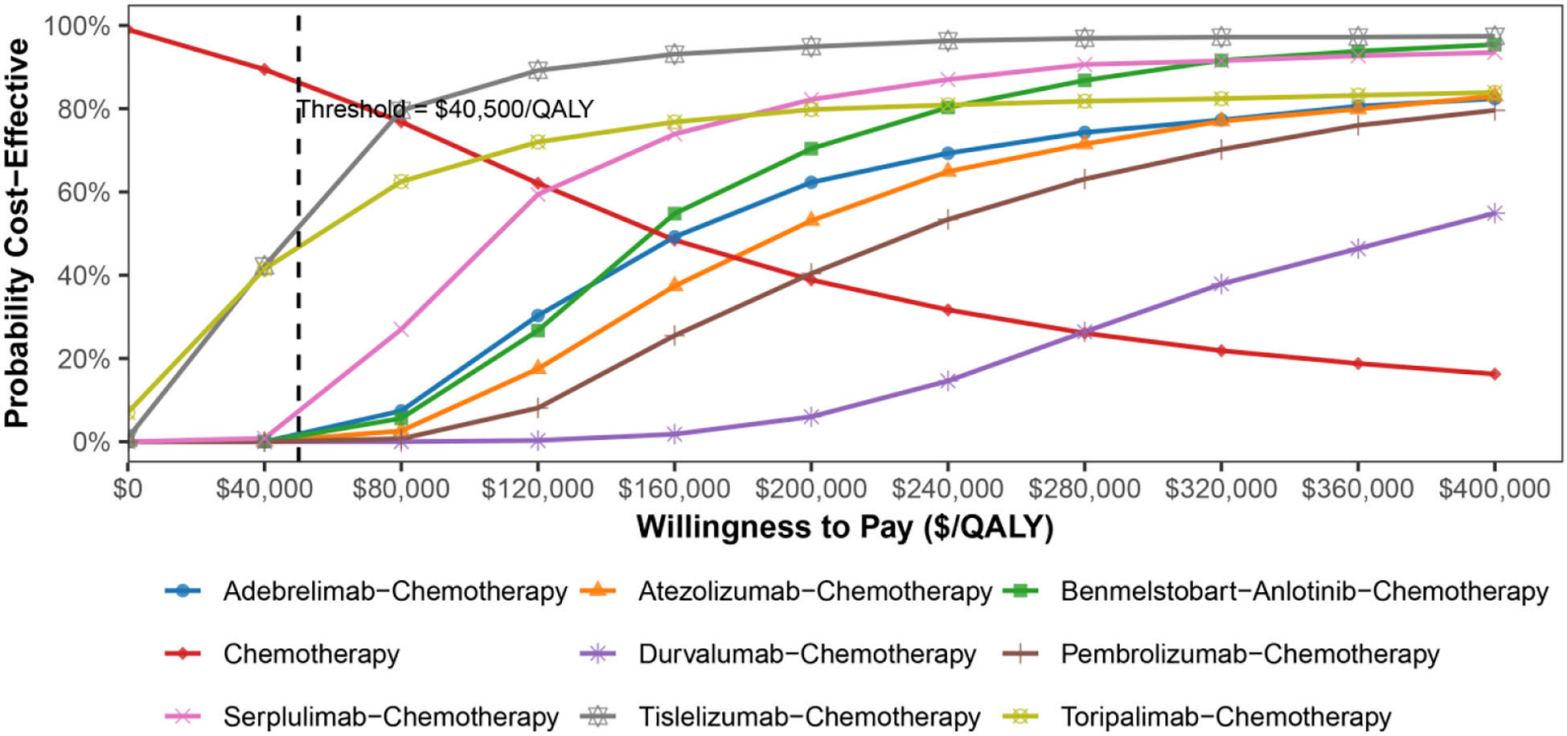
Cost-effectiveness acceptability curves of eight ICI-based regimens and chemotherapy alone in ES-SCLC patients.

The cost-effectiveness scatter plot and acceptability curve (CEAC) are presented in **Figures 5**, **6**. PSA (Monte Carlo simulation, 1,000 iterations) confirmed that at a $40,500/QALY threshold, none of the ICIs-based regimens was cost-effective as compared to chemotherapy alone. However, tislelizumab and toripalimab demonstrated relative economic advantages compared to other treatments. CEAC indicated that when the WTP threshold increased to $80,000 per QALY, the combination of chemotherapy with tislelizumab demonstrated a >70% probability of being cost-effective; it continued to increase with further WTP threshold increments, emerging as the preferred option. However, at a WTP threshold of $40,500 per QALY, none of the eight treatment regimens showed cost-effectiveness as compared to chemotherapy alone, confirming the robustness of the base-case analysis results.

## 4 Discussion

The emergence of ICIs has dramatically transformed the treatment landscape for patients with ES-SCLC. Numerous studies have demonstrated that the combination of ICIs with chemotherapy, particularly triplet combinations of ICIs, chemotherapy, and anti-angiogenic agents, can synergistically inhibit tumor growth by suppressing angiogenesis and controlling tumor cell proliferation and metastasis, thus ultimately improving the OS and PFS of patients (34, 35). However, given the substantial economic burden associated with the high-cost combination regimens, the optimal balance among efficacy, safety, and cost-effectiveness for ES-SCLC treatment remains unclear. This study represents the first systematic comparison to determine the superior therapeutic regimen based on the comprehensive evaluations of clinical efficacy, safety profiles, and economic outcomes.

Efficacy analyses revealed that the combination of benmelstobart and anlotinib with chemotherapy significantly improved OS, markedly prolonging PFS as compared to chemotherapy alone as well as all other ICI-based regimens. NMA did not reveal statistically significant differences in OS or ORR between the benmelstobart and other ICI regimens; however, benmelstobart consistently demonstrated favorable trends in prolonging disease control and reducing disease progression risk (all HRs <1, all ORR >1), suggesting potential clinical benefits. The treatment rankings based on SUCRA values further confirmed the therapeutic superiority of benmelstobart in terms of OS, PFS, and ORR. On the other hand, benmelstobart significantly increased the incidence of ≥grade 3 AEs as compared to chemotherapy alone. The comparisons among ICI regimens showed no statistically significant differences in high-grade AE rates, except for benmelstobart, which exhibited a consistently higher incidence trend (all OR >1). On the other hand, tislelizumab demonstrated a relatively favorable safety profile (all OR <1), suggesting that the combination of tislelizumab and chemotherapy might offer clinical advantages regarding toxicity management. Among the assessed outcomes, heterogeneity was notably high for PFS, whereas OS, ORR, and AE outcomes showed relatively low heterogeneity. A comprehensive evaluation of methodological, clinical, and statistical factors revealed no significant differences in study design, participant characteristics, or randomization methods. Detailed analyses indicated that the heterogeneity was predominantly driven by two trials involving serplulimab and benmelstobart, largely due to their pronounced PFS improvements. These substantial benefits widened the disparity in effect estimates across studies, thereby contributing substantially to the observed heterogeneity an d necessitating the application of a random-effects model.

In light of recent advances in SCLC, our findings merit further contextualization. Although adding PD-1/PD-L1 inhibitors to platinum–etoposide chemotherapy has become the new first-line standard, the overall survival gain remains modest (9–14, 16). Consequently, recent studies have begun to explore triplet regimens that combine immunotherapy and chemotherapy with additional strategies, such as anti-angiogenic therapy, radiotherapy, or novel investigational agents (15, 36–39). Notably, the ETER701 trial demonstrated unprecedented improvements in OS, PFS, and ORR for ES-SCLC with the addition of benmelstobart plus anlotinib, thereby offering a promising new option for first-line treatment (15). Our NMA produced consistent results, showing that benmelstobart exhibited the greatest clinical benefit compared to other ICI-based regimens. These benefits likely reflect the dual mechanism of PD-L1 blockade restoring T-cell activity and anti-angiogenic agents suppressing angiogenic signaling, which synergistically enhance antitumor activity (40, 41). However, these survival gains come at the expense of increased toxicity, as our NMA also showed that benmelstobart was consistently associated with higher rates of grade ≥3 adverse events. Such differences may also relate to molecular subtypes and biomarkers, but no robust predictive biomarker has yet been validated for clinical use in SCLC (42). Future research should therefore focus on identifying effective biomarkers to precisely identify patients most likely to benefit and refining combination strategies to maximize efficacy while minimizing toxicity.

Nevertheless, none of the eight ICI-based regimens were cost-effective under the predefined WTP threshold of $40,500/QALY. While chemotherapy alone remained cost-effective, it offered limited clinical benefit. Despite benmelstobart providing the highest QALYs at 1.31, its substantial cost ($102,934.50) increased the ICER, thus diminishing its overall cost-effectiveness. On the other hand, tislelizumab, achieving 1.27 QALYs at a comparatively lower cost ($52,273.46), emerged as the most economically favorable option among ICI-based therapies. Sensitivity analyses highlighted that the key factors affecting ICER estimates included drug costs and health state values for PFS. In PSA, tislelizumab and toripalimab showed measurable economic superiority as compared to other ICI-based regimens at the WTP threshold of $40,500/QALY. According to the CEAC, when the WTP threshold was increased to $80,000 per QALY, tislelizumab emerged as the most economically favorable treatment option, surpassing a 70% probability of cost-effectiveness. With a further increase in the WTP threshold, the cost-effectiveness probability of tislelizumab progressively approached nearly 100%. The combination of chemotherapy with benmelstobart and anlotinib and the combination of chemotherapy with serplulimab also exhibited improved economic viability at higher thresholds, which might be attributed to their high QALYs. Altogether, this study found that although certain combination therapies demonstrated survival benefits, their substantial additional costs outweigh the marginal clinical advantages, rendering the first-line immuno-chemotherapy potentially unfavorable from a cost-effectiveness perspective in China.

This study possesses several notable strengths. First, it presented a comprehensive comparative assessment that simultaneously attempted to address the efficacy, safety, and cost-effectiveness of all first-line ICI-chemotherapy combinations recommended by the Chinese Society of Clinical Oncology (CSCO) 2025 guidelines for ES-SCLC (20). Moreover, the newly approved benmelstobart regimen was also included, thereby enriching the latest evidence aligned with current clinical practice (43). Second, acknowledging the inherently limited duration of clinical trials, appropriate survival curve extrapolation techniques were employed to estimate long-term clinical outcomes, thereby enhancing the generalizability and practical applicability of the findings. Third, distinct from prior investigations, the current study results identified tislelizumab as the most cost-effective first-line therapeutic option among ICI-based regimens for ES-SCLC, providing a clinically effective and economically viable alternative, which might be particularly beneficial for patients facing financial constraints (43, 44). Moreover, by implementing the rigorously standardized platinum-based chemotherapy controls and strictly adhering to clinical trial protocols, this study ensured a high degree of credibility as compared to previous studies (45). Consequently, this study provided comprehensive insights by evaluating the cost-effectiveness, clinical effectiveness, and safety profiles of eight ICI-chemotherapy combinations as compared to chemotherapy alone, as well as among the eight ICI-based regimens. Thus, this study offers valuable evidence-based insights for clinicians, facilitating more informed therapeutic decisions by balancing clinical effectiveness, toxicity, and economic considerations.

Nonetheless, the present study has several limitations. First, our NMA included four PD-1 and four PD-L1 inhibitors, which were analyzed within a single network. Given the ongoing scientific debate regarding potential efficacy or safety differences between these two classes of inhibitors, pooling them may have obscured class-specific effects. Second, only grade ≥ 3 AEs were included, while lower-grade AEs were omitted, potentially biasing the ICERs. Moreover, disutility values for AEs were not incorporated into the model. Consequently, the detrimental impact of treatment-related toxicities on quality of life was not captured, which may have led to underestimation of the ICERs and thus an overestimation of the cost-effectiveness of certain regimens. Third, in the absence of direct quality-of-life and cost data, health-state utilities and cost inputs were drawn from published literature, thereby introducing potential uncertainty. Finally, the patients entering the PD health state were assumed to receive the same subsequent therapy, and the costs for follow-up testing, subsequent therapy, BSC, and AE management were assumed to be uniform across all eight trials. These assumptions do not capture real-world variations in medication regimens, which might lead to inaccurate cost estimations.

## 5 Conclusions

In conclusion, this study demonstrated that the combination of chemotherapy with benmelstobart and anlotinib provided the greatest clinical benefit regarding OS, PFS, and ORR, followed by the combination of chemotherapy and serplulimab. Moreover, the combination of chemotherapy and tislelizumab provided the most favorable safety profile among the evaluated ICI-based therapies. From the perspective of the Chinese healthcare system, none of the eight immuno-chemotherapy regimens were cost-effective at the conventional WTP threshold ($40,500/QALY) as compared to chemotherapy alone. However, the combination of chemotherapy and tislelizumab was the most cost-effective treatment as the WTP threshold increased. These findings provide essential evidence for clinicians, aiding in selecting effective, safe, and economically justified treatment strategies for patients with ES-SCLC.

## Data Availability

The original contributions presented in the study are included in the article/**Supplementary Material**. Further inquiries can be directed to the corresponding author.
