# Supplementary material for: Immune checkpoint inhibitors for extensive-stage small-cell lung cancer: a network meta-analysis and cost-effectiveness analysis
Source: Front Immunol. 2025 Oct 27;16:1662438. doi: 10.3389/fimmu.2025.1662438 (PMC12597965; doi:10.3389/fimmu.2025.1662438)
Supplement: Supplementary file 1 [file DataSheet1.docx]

Supplementary Material

**Supplementary Figure S1.** Reconstructed Kaplan-Meier plots of overall survival.

**Supplementary Figure S2.** Reconstructed Kaplan-Meier plots of progression-free survival.

**Supplementary Figure S3.** The fitting curve for extrapolating the original K-M curve over a ten-year period of overall survival.

**Supplementary Figure S4.** The fitting curve for extrapolating the original K-M curve over a ten-year period of progression-free survival.

**Supplementary Figure S5.** Study selection.

**Supplementary Figure S6.** Model diagram of network meta - analysis.

**Supplementary Figure S7.** Assessment of risk of bias.

**Supplementary Table S1.** PRISMA NMA Checklist.

**Supplementary Table S2.** Search strategy.

**Supplementary Table S3.** Characteristics of RCTs included in the study.

**Supplementary Table S4.** CHEERS Checklist.

**Supplementary Table S5.** Summary of statistical goodness-of-fit of K-M curve.

**Supplementary Table S6.** Optimal Fitting Distributions and Parameters.

**Supplementary Table S7.** Model Parameters: Clinical and Cost data.


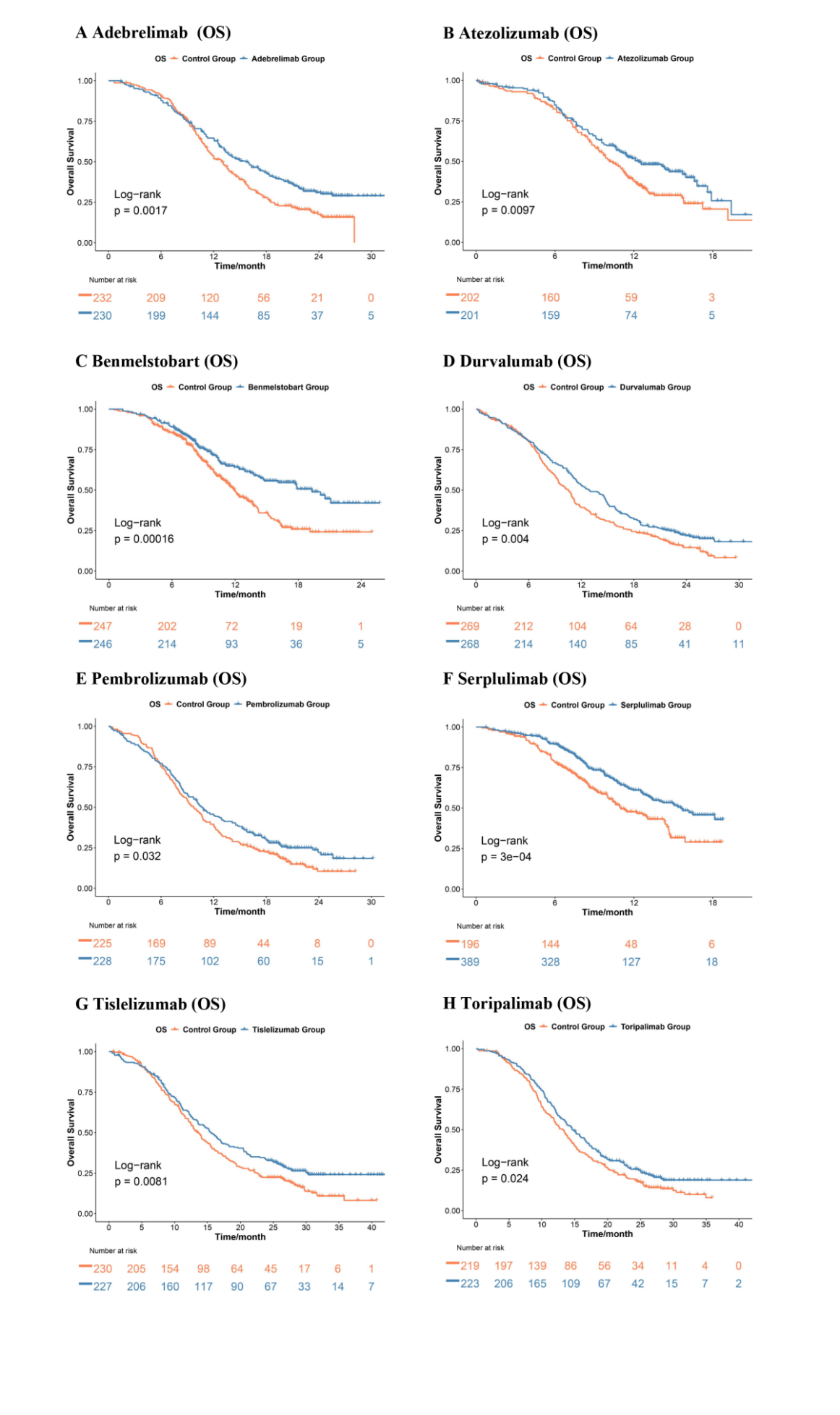


**Supplementary Figure S1.** Reconstructed Kaplan-Meier plots of overall survival. (A) Adebrelimab, (B) Atezolizumab, (C) Benmelstobart, (D) Durvalumab, (E) Pembrolizumab, (F) Serplulimab, (G) Tislelizumab, and (H) Toripalimab.


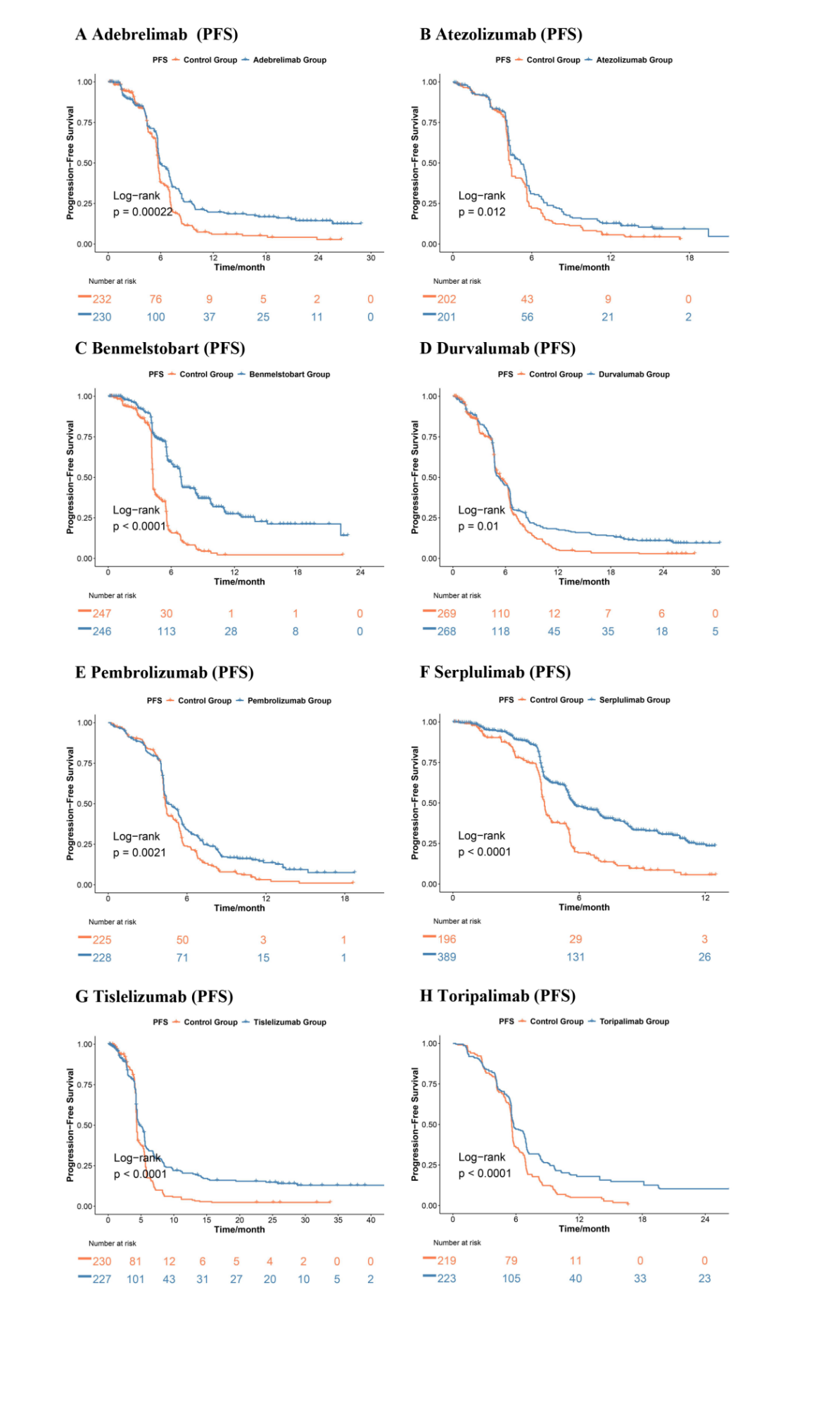


**Supplementary Figure S2.** Reconstructed Kaplan-Meier plots of progression-free survival. (A) Adebrelimab, (B) Atezolizumab, (C) Benmelstobart, (D) Durvalumab, (E) Pembrolizumab, (F) Serplulimab, (G) Tislelizumab, and (H) Toripalimab.


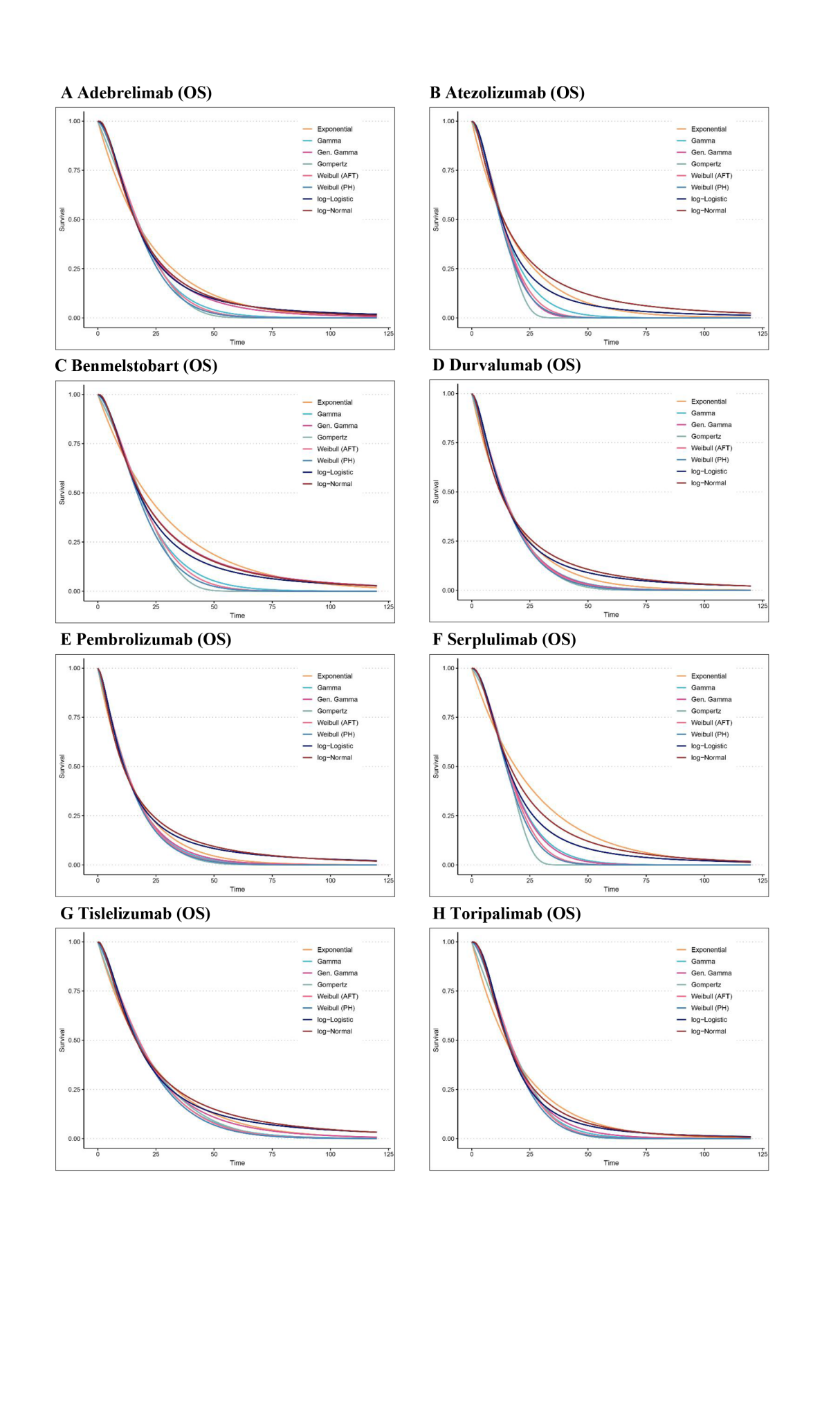


**Supplementary Figure S3.** The fitting curve for extrapolating the original K-M curve over a ten-year period of overall survival. (A) Adebrelimab, (B) Atezolizumab, (C) Benmelstobart, (D) Durvalumab, (E) Pembrolizumab, (F) Serplulimab, (G) Tislelizumab, (H) Toripalimab.


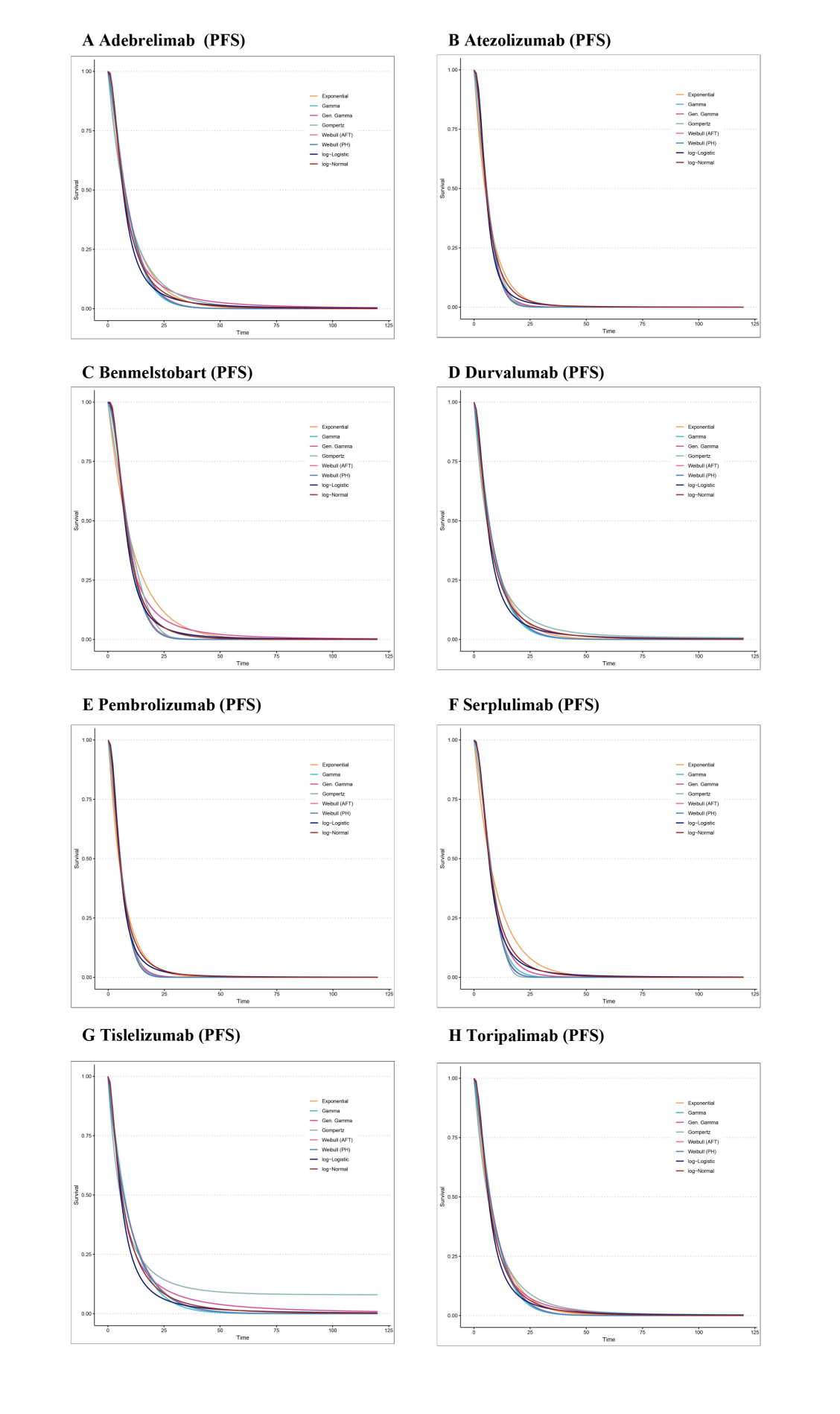


**Supplementary Figure S4.** The fitting curve for extrapolating the original K-M curve over a ten-year period of progression-free survival. (A) Adebrelimab, (B) Atezolizumab, (C) Benmelstobart, (D) Durvalumab, (E) Pembrolizumab, (F) Serplulimab, (G) Tislelizumab, (H) Toripalimab.


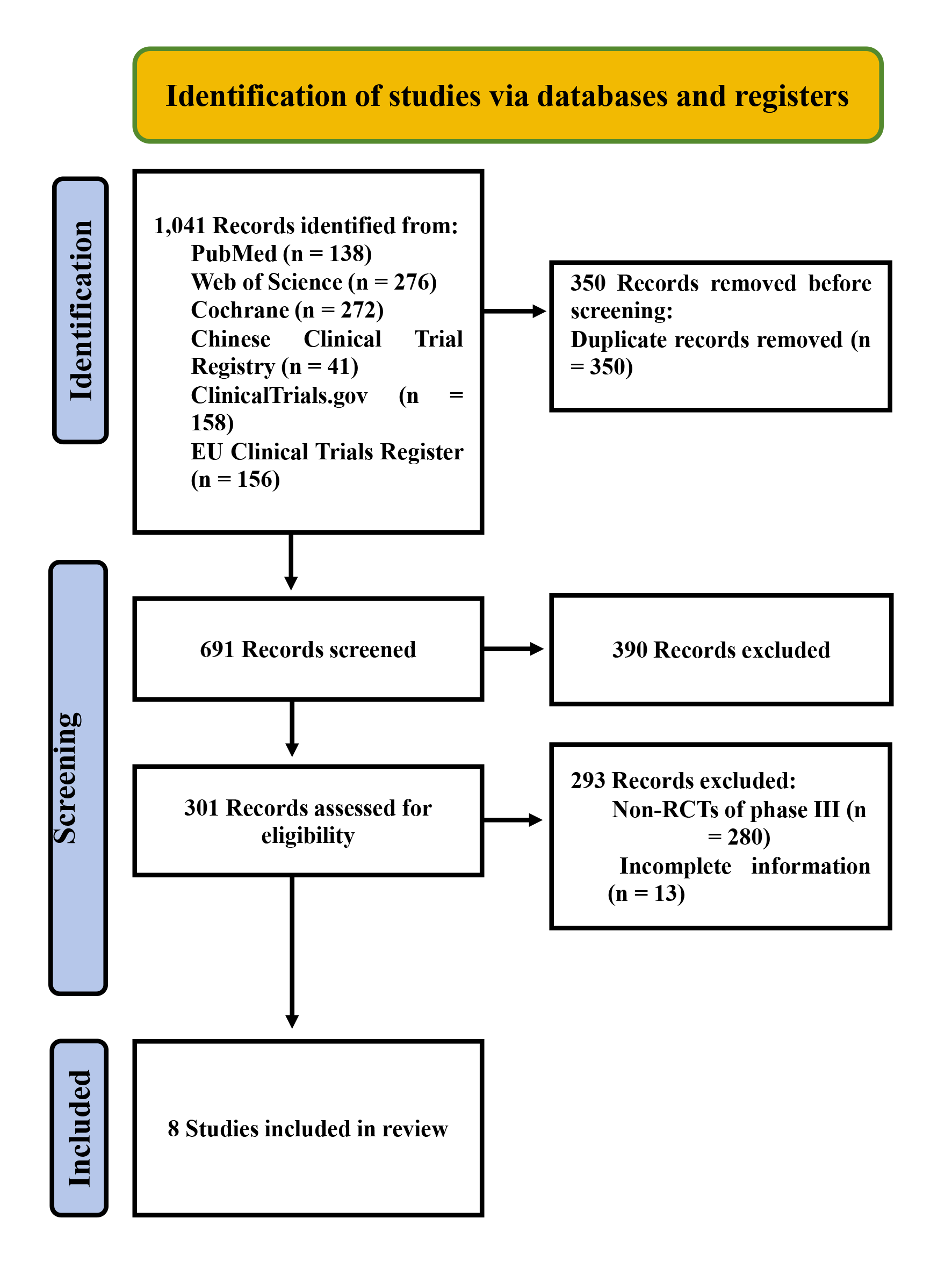


**Supplementary Figure S5.** Study selection.


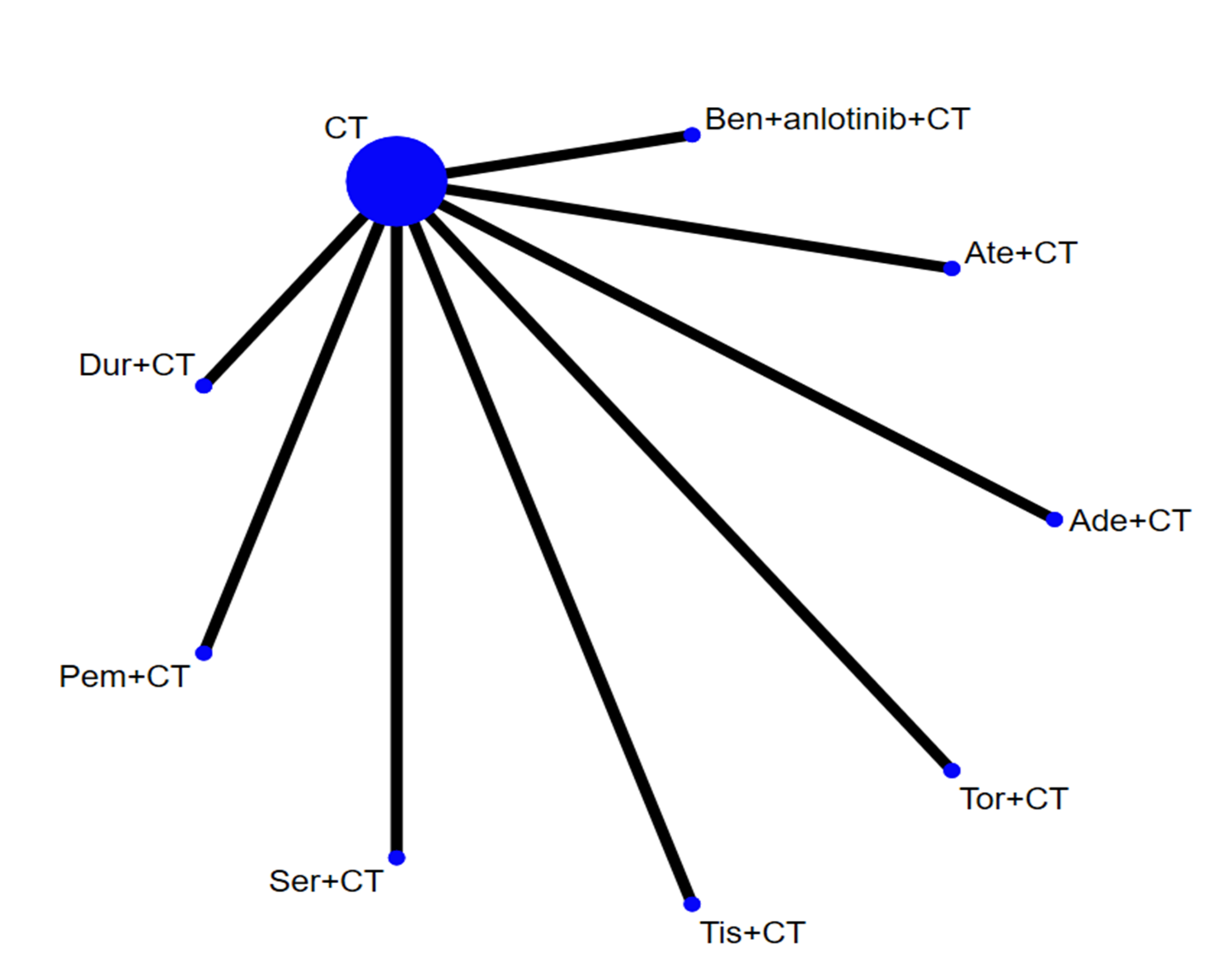


**Supplementary Figure S6.** Model diagram of network meta - analysis. CT, chemotherapy; Ate, atezolizumab; Dur, durvalumab; Ser, Serplulimab; Ade, adebrelimab; Pem, Pembrolizumab; Ben, Benmelstobart; Tis, Tislelizumab; Tor, Toripalimab.


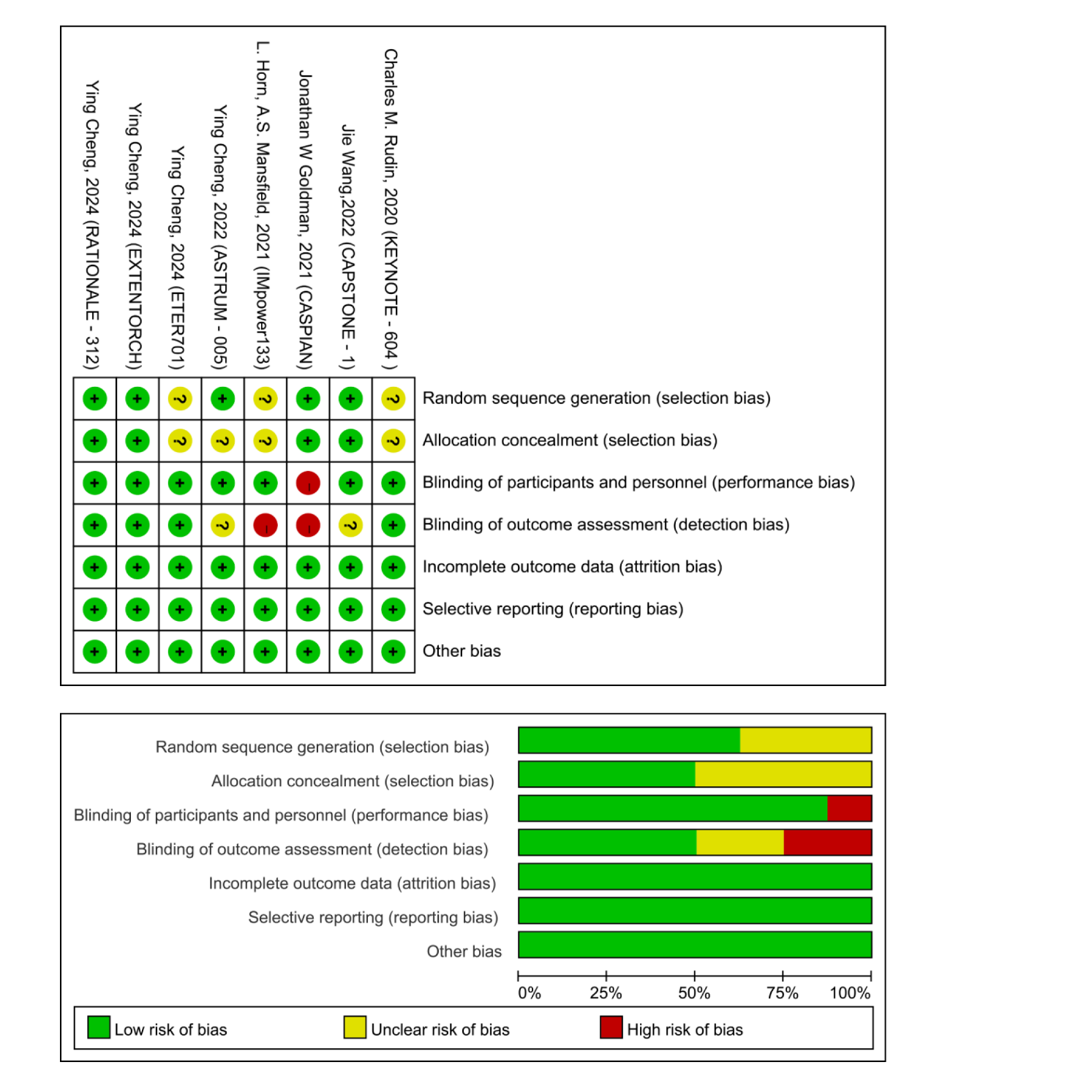


**Supplementary Figure S7.** Assessment of risk of bias.

# Supplementary Table S1. PRISMA NMA Checklist.

| Section and Topic | Item # | Checklist item | Location where item is reported |
| --- | --- | --- | --- |
| TITLE | | | |
| Title | 1 | Identify the report as a systematic review incorporating a network meta - analysis (or related form of meta - analysis) | 1 |
| ABSTRACT | | | |
| Abstract | 2 | Provide a structured summary including, as applicable:  **Background:** main objectives  **Methods:** data sources; study eligibility criteria, participants, and interventions; study appraisal; and synthesis methods, such as network meta - analysis.  **Results:** number of studies and participants identified; summary estimates with corresponding confidence/credible intervals; treatment rankings may also be discussed. Authors may choose to summarize pairwise comparisons against a chosen treatment included in their analyses for brevity.  **Discussion/Conclusions:** limitations; conclusions and implications of findings.  **Other:** primary source of funding; systematic review registration number with registry name. | 1 |
| INTRODUCTION | | | |
| Rationale | 3 | Describe the rationale for the review in the context of existing knowledge. | 2 |
| Objectives | 4 | Provide an explicit statement of the objective (s) or question (s) the review addresses. | 2-3 |
| METHODS | | | |
| Eligibility criteria | 5 | Specify the inclusion and exclusion criteria for the review and how studies were grouped for the syntheses. | 3 |
| Information sources | 6 | Specify all databases, registers, websites, organisations, reference lists and other sources searched or consulted to identify studies. Specify the date when each source was last searched or consulted. | 2-3 |
| Search strategy | 7 | Present the full search strategies for all databases, registers and websites, including any filters and limits used. | Supplementary Table S2 |
| Selection process | 8 | Specify the methods used to decide whether a study met the inclusion criteria of the review, including how many reviewers screened each record and each report retrieved, whether they worked independently, and if applicable, details of automation tools used in the process. | 3 |
| Data collection process | 9 | Specify the methods used to collect data from reports, including how many reviewers collected data from each report, whether they worked independently, any processes for obtaining or confirming data from study investigators, and if applicable, details of automation tools used in the process. | 2-3 |
| Data items | 10a | List and define all outcomes for which data were sought. Specify whether all results that were compatible with each outcome domain in each study were sought (e.g. for all measures, time points, analyses), and if not, the methods used to decide which results to collect. | 3 |
|  | 10b | List and define all other variables for which data were sought (e.g. participant and intervention characteristics, funding sources). Describe any assumptions made about any missing or unclear information. | Supplementary Table S3 |
| Study risk of bias assessment | 11 | Specify the methods used to assess risk of bias in the included studies, including details of the tool (s) used, how many reviewers assessed each study and whether they worked independently, and if applicable, details of automation tools used in the process. | 3 |
| Effect measures | 12 | Specify for each outcome the effect measure (s) (e.g. risk ratio, mean difference) used in the synthesis or presentation of results. | Supplementary Table S3 |
| Synthesis methods | 13a | Describe the processes used to decide which studies were eligible for each synthesis (e.g. tabulating the study intervention characteristics and comparing against the planned groups for each synthesis (item #5)). | Supplementary Figure S6  Supplementary Table S3 |
|  | 13b | Describe any methods required to prepare the data for presentation or synthesis, such as handling of missing summary statistics, or data conversions. | 3 |
|  | 13c | Describe any methods used to tabulate or visually display results of individual studies and syntheses. | 3 |
|  | 13d | Describe any methods used to synthesize results and provide a rationale for the choice (s). If meta-analysis was performed, describe the model (s), method (s) to identify the presence and extent of statistical heterogeneity, and software package (s) used. | 3 |
|  | 13e | Describe any methods used to explore possible causes of heterogeneity among study results (e.g. subgroup analysis, meta-regression). | 7 |
|  | 13f | Describe any sensitivity analyses conducted to assess robustness of the synthesized results. | Not applicable |
| Reporting bias assessment | 14 | Describe any methods used to assess risk of bias due to missing results in a synthesis (arising from reporting biases). | Not applicable |
| Certainty assessment | 15 | Describe any methods used to assess certainty (or confidence) in the body of evidence for an outcome. | 3 |
| RESULTS | | | |
| Study selection | 16a | Describe the results of the search and selection process, from the number of records identified in the search to the number of studies included in the review, ideally using a flow diagram. | Supplementary Figure S5 |
|  | 16b | Cite studies that might appear to meet the inclusion criteria, but which were excluded, and explain why they were excluded. | Not applicable |
| Study characteristics | 17 | Cite each included study and present its characteristics. | Supplementary Table S3 |
| Risk of bias in studies | 18 | Present assessments of risk of bias for each included study. | Supplementary Figure S7 |
| Results of individual studies | 19 | For all outcomes, present, for each study: (a) summary statistics for each group (where appropriate) and (b) an effect estimate and its precision (e.g. confidence/credible interval), ideally using structured tables or plots. | Figure 2 |
| Results of syntheses | 20a | For each synthesis, briefly summarise the characteristics and risk of bias among contributing studies. | 5  Supplementary Figure S7 |
|  | 20b | Present results of all statistical syntheses conducted. If meta-analysis was done, present for each the summary estimate and its precision (e.g. confidence / credible interval) and measures of statistical heterogeneity. If comparing groups, describe the direction of the effect. | Figure 2 |
|  | 20c | Present results of all investigations of possible causes of heterogeneity among study results. | 7 |
|  | 20d | Present results of all sensitivity analyses conducted to assess the robustness of the synthesized results. | Not applicable |
| Reporting biases | 21 | Present assessments of risk of bias due to missing results (arising from reporting biases) for each synthesis assessed. | Not applicable |
| Certainty of evidence | 22 | Present assessments of certainty (or confidence) in the body of evidence for each outcome assessed. | Figure 2  Figure 3 |
| DISCUSSION |  |  |  |
| Discussion | 23a | Provide a general interpretation of the results in the context of other evidence. | 7-8 |
|  | 23b | Discuss any limitations of the evidence included in the review. | 11 |
|  | 23c | Discuss any limitations of the review processes used. | 11 |
|  | 23d | Discuss implications of the results for practice, policy, and future research. | 11 |

***The PRISMA extension checklist was developed for RICE-META1st based on the structure of the PRISMA Network Meta-Analysis (NMA) reporting checklist, accessible at** <https://www.prisma-statement.org/nma>(1)**.**

**Supplementary Table S2.** Search strategy.

| Database | Keywords |
| --- | --- |
| PubMed | 138 |
| (1) | “Small Cell Lung Carcinoma”[MeSH Terms] OR “Carcinoma, Small Cell Lung”[Title/Abstract] OR “Oat Cell Carcinoma of Lung”[Title/Abstract] OR “Oat Cell Lung Cancer”[Title/Abstract] OR “Small Cell Cancer Of The Lung”[Title/Abstract] OR “Small Cell Lung Cancer”[Title/Abstract] |
| (2) | “durvalumab”[MeSH Terms] OR “MEDI4736”[Title/Abstract] OR “MEDI-4736”[Title/Abstract] OR “Imfinzi”[Title/Abstract] |
| (3) | “atezolizumab”[MeSH Terms] OR “anti-PDL1”[Title/Abstract] OR “MPDL3280A”[Title/Abstract] OR “MPDL-3280A”[Title/Abstract] OR “Tecentriq”[Title/Abstract] OR “RG7446”[Title/Abstract] OR “RG-7446”[Title/Abstract] |
| (4) | “serplulimab”[Title/Abstract] OR “adebrelimab”  [Title/Abstract] OR “benmelstobart” [Title/Abstract] OR “toripalimab”  [Title/Abstract] |
| (5) | “pembrolizumab”[MeSH Terms] OR “MK-3475”[Title/Abstract] OR “Keytruda”[Title/Abstract] OR “lambrolizumab”[Title/Abstract] OR “SCH-900475”[Title/Abstract] |
| (6) | “tislelizumab”[MeSH Terms] OR “BGB-A317”[Title/Abstract] |
| (7) | “clinical trials as topic” OR “trial” OR “phase III” OR “phase 3” OR “clinical trials” |
| (8) | ((2)OR (3) OR (4) OR (5) OR (6) )AND (1) AND (7) |
| (9) | “Carcinoma, Non-Small-Cell Lung”[MeSH Terms] OR “Carcinoma, Non Small Cell Lung”[Title/Abstract] OR “Carcinomas, Non-Small-Cell Lung”[Title/Abstract] OR “Lung Carcinoma, Non-Small-Cell”[Title/Abstract] OR “Lung Carcinomas, Non-Small-Cell”[Title/Abstract] OR “Non-Small-Cell Lung Carcinomas”[Title/Abstract] OR “Carcinoma, Non-Small Cell Lung”[Title/Abstract] OR “Non-Small Cell Lung Cancer”[Title/Abstract] OR “Non-Small-Cell Lung Carcinoma”[Title/Abstract] OR “Non Small Cell Lung Carcinoma”[Title/Abstract] OR “Non-small Cell Lung Cancer”[Title/Abstract] OR “Non-Small Cell Lung Carcinoma”[Title/Abstract] |
| (10) | “meta”[Title] OR “meta-analysis”[Title] OR “review”[Title] OR “protocol”[Title] |
| (11) | 1. NOT (9) NOT (10) |
| Web of science | 276 |
| (1) | TS=('Small Cell Lung Carcinoma' OR 'Carcinoma, Small Cell Lung' OR 'Oat Cell Carcinoma of Lung' OR 'Oat Cell Lung Cancer' OR 'Small Cell Cancer Of The Lung' OR 'Small Cell Lung Cancer') |
| (2) | TS=(durvalumab OR MEDI4736 OR MEDI-4736 OR Imfinzi) |
| (3) | TS=(atezolizumab OR anti-PDL1 OR MPDL3280A OR MPDL-3280A OR Tecentriq OR RG7446 OR RG-7446) |
| (4) | TS=(serplulimab OR adebrelimab OR benmelstobart OR toripalimab) |
| (5) | TS=(pembrolizumab OR MK-3475 OR Keytruda OR lambrolizumab OR SCH-900475) |
| (6) | TS=(tislelizumab OR BGB-A317) |
| (7) | TS=('Carcinoma, Non-Small-Cell Lung' OR 'Carcinoma, Non Small Cell Lung' OR 'Carcinomas, Non-Small-Cell Lung' OR 'Lung Carcinoma, Non-Small-Cell' OR 'Lung Carcinomas, Non-Small-Cell' OR 'Non-Small-Cell Lung Carcinomas' OR 'Carcinoma, Non-Small Cell Lung' OR 'Non-Small Cell Lung Cancer' OR 'Non-Small-Cell Lung Carcinoma' OR 'Non Small Cell Lung Carcinoma' OR 'Non-small Cell Lung Cancer' OR 'Non-Small Cell Lung Carcinoma') |
| (8) | TS=(conference OR review OR meta) |
| (9) | TS=('clinical trials as topic' OR trial OR 'phase III' OR 'phase 3' OR 'clinical trials') |
|  | (((2)OR(3)OR(4)OR(5)OR(6))AND(1)AND(9))NOT(8)NOT(7) |
| Cochrane | 272 |
| (1) | ('Small Cell Lung Carcinoma' OR 'Carcinoma, Small Cell Lung' OR 'Oat Cell Carcinoma of Lung' OR 'Oat Cell Lung Cancer' OR 'Small Cell Cancer Of The Lung' OR 'Small Cell Lung Cancer'):ab,ti,kw |
| (2) | (durvalumab OR MEDI4736 OR MEDI-4736 OR Imfinzi):ab,ti,kw |
| (3) | (atezolizumab OR anti-PDL1 OR MPDL3280A OR MPDL-3280A OR Tecentriq OR RG7446 OR RG-7446):ab,ti,kw |
| (4) | (serplulimab OR adebrelimab OR benmelstobart OR toripalimab):ab,ti,kw |
| (5) | (pembrolizumab OR MK-3475 OR Keytruda OR lambrolizumab OR SCH-900475):ab,ti,kw |
| (6) | (tislelizumab OR BGB-A317):ab,ti,kw |
| (7) | ('Carcinoma, Non-Small-Cell Lung' OR 'Carcinoma, Non Small Cell Lung' OR 'Carcinomas, Non-Small-Cell Lung' OR 'Lung Carcinoma, Non-Small-Cell' OR 'Lung Carcinomas, Non-Small-Cell' OR 'Non-Small-Cell Lung Carcinomas' OR 'Carcinoma, Non-Small Cell Lung' OR 'Non-Small Cell Lung Cancer' OR 'Non-Small-Cell Lung Carcinoma' OR 'Non Small Cell Lung Carcinoma' OR 'Non-small Cell Lung Cancer' OR 'Non-Small Cell Lung Carcinoma'):ab,ti,kw |
| (8) | ('clinical trials as topic' OR trial OR 'phase III' OR 'phase 3' OR 'clinical trials'):ab,ti,kw |
| (9) | ((2)OR (3) OR (4) OR (5) OR (6) )AND (1) AND (8) NOT (7) |
| Chinese Clinical Trial Registry | 41 |
| (1) | “durvalumab”,“atezolizumab”,“serplulimab”,“pembrolizumab”,“tislelizumab”,“benmelstobart”, “toripalimab”, “adebrelimab” |
| ClinicalTrials.gov | 158 |
| (1) | “durvalumab”, “atezolizumab”, “serplulimab”, “pembrolizumab”, “tislelizumab”, “benmelstobart”, “toripalimab”, “adebrelimab” |
| European Union Clinical Trials Register | 156 |
| (1) | “durvalumab”, “atezolizumab”, “serplulimab”, “pembrolizumab”, “tislelizumab”, “benmelstobart”, “toripalimab”, “adebrelimab” |

**Supplementary Table S3.** Characteristics of RCTs included in the study.

| Study | Trial name | Intervention  arm | Sample size | ORR (%) | Control  arm | Sample size | ORR (%) | OS (months) | HR for OS (95% CI) | PFS (months) | HR for PFS (95% CI) | Patients with grade 3 or higher AEs (%) | Incorporate outcome indicators |
| --- | --- | --- | --- | --- | --- | --- | --- | --- | --- | --- | --- | --- | --- |
| Jie Wang,  2022 | CAPSTONE-1 | Adebrelimab + Chemotherapy | 230 | 70.4 | Chemotherapy | 232 | 65.9 | 15.3 vs 12.8 | 0.72 (0.58-0.90); | 5.8 vs 5.6 | 0.67 (0.54-0.83) | 85.7 vs 84.9 | OS, PFS, ORR, ≥grade 3 AEs (%) |
| Charles M. Rudin, 2020 | KEYNOTE-604 | Pembrolizumab + Chemotherapy | 228 | 70.6 | Chemotherapy | 225 | 61.8 | 10.8 vs 9.7 | 0.80 (0.64-0.98) | 4.5 vs 4.3 | 0.75 (0.61-0.91) | 76.7 vs 74.9 (grade 3+grade 4) | OS, ORR, PFS, ≥grade 3 AEs (%) |
| Ying Cheng, 2022 | ASTRUM-005 | Serplulimab + Chemotherapy | 389 | 80.2 | Chemotherapy | 196 | 70.4 | 15.4 vs 10.9 | 0.63 (0.49-0.82) | 5.7 vs 4.3 | 0.48 (0.38-0.59) | 33.2 vs 27.6 | OS, PFS, ORR, ≥grade 3 AEs (%) |
| Ying Cheng, 2024 | RATIONALE-312 | Tislelizumab + Chemotherapy | 227 | 68.0 | Chemotherapy | 230 | 62.0 | 15.5 vs 13.5 | 0.75 (0.61-0.93) | 4.7 vs 4.3 | 0.64 (0.52-0.78) | 85.5 vs 86.0 | OS, PFS, ORR, ≥grade 3 AEs (%) |
| L. Horn, A.S. Mansfield, 2021 | IMpower133 | Atezolizumab + Chemotherapy | 201 | 60.2 | Chemotherapy | 202 | 64.4 | 12.3 vs 10.3 | 0.76 (0.60-0.95) | 5.2 vs 4.3 | 0.77 (0.63-0.95) | 58.6 vs 57.7 | OS, PFS, ORR, ≥grade 3 AEs (%) |
| Jonathan W Goldman, 2021 | CASPIAN | Durvalumab + Chemotherapy | 268 | 67.9 | Chemotherapy | 269 | 58.0 | 12.9 vs 10.5 | 0.75 (0.62-0.91) | 5.1 vs 5.4 | 0.80 ( 0.66-0.96) | 64.5 vs 65.0 | OS, PFS, ORR, ≥grade 3 AEs (%) |
| Ying Cheng, 2024 | ETER701 | Benmelstobart + Anlotinib + Chemotherapy | 246 | 81.3 | Chemotherapy | 247 | 66.8 | 19.3 vs 11.9 | 0.61 (0.47-0.79) | 6.9 vs 4.2 | 0.32 (0.26-0.41) | 93.1 vs 87.0 | OS, PFS, ORR, ≥grade 3 AEs (%) |
| Ying Cheng, 2024 | EXTENTORCH | Toripalimab + Chemotherapy | 223 | 78.0 | Chemotherapy | 219 | 73.1 | 14.6 vs 13.3 | 0.80 (0.65-0.98) | 5.8 vs 5.6 | 0.67 (0.54-0.82) | 89.6 vs 89.4 | OS, PFS, ORR, ≥grade 3 AEs (%) |

**Supplementary Table S4.** CHEERS Checklist.

| Section/item | | Item No | Recommendation | | Reported on page No |
| --- | --- | --- | --- | --- | --- |
| Title and abstract | | |  | |  |
| Title | | 1 | Identify the study as an economic evaluation or use more specific terms such as “cost-effectiveness analysis”, and describe the interventions compared. | | 1 |
| Abstract | | 2 | Provide a structured summary of objectives, perspective, setting, methods (including study design and inputs), results (including base case and uncertainty analyses), and conclusions. | | 1 |
| **Introduction** | | |  | |  |
| Background and  objectives | | 3 | Provide an explicit statement of the broader context for the study. | | 2-3 |
|  |  |  | Present the study question and its relevance for health policy or practice decisions. | |  |
| **Methods** | | |  | |  |
| Target population and subgroups | | 4 | Describe characteristics of the base case population and subgroups analysed, including why they were chosen. | | Supplementary Table S3 |
| Setting and  location | | 5 | State relevant aspects of the system(s) in which the decision(s) need(s) to be made. | | 3-4 |
| Study perspective | | 6 | Describe the perspective of the study and relate this to the costs being evaluated. | | 4 |
| Comparators | | 7 | Describe the interventions or strategies being compared and state why they were chosen. | | 3 |
| Time horizon | | 8 | State the time horizon(s) over which costs and consequences are being evaluated and say why appropriate. | | 3 |
| Discount rate | | 9 | Report the choice of discount rate(s) used for costs and outcomes and say why appropriate. | | 4 |
| Choice of health outcomes | | 10 | Describe what outcomes were used as the measure(s) of benefit in the evaluation and their relevance for the type of analysis performed. | | 4-5 |
| Measurement of  effectiveness | | 11a | *Single study-based estimates:* Describe fully the design features of the single effectiveness study and why the single study was a sufficient source of clinical effectiveness data. | | 5 |
|  | | 11b | *Synthesis-based estimates:* Describe fully the methods used for identification of included studies and synthesis of clinical effectiveness data. | | 3-4 |
| Measurement valuation preference outcomes | and of based | 12 | If applicable, describe the population and methods used to elicit preferences for outcomes. | | 3 |
| Estimating resources costs | and | 13a | *Single study-based economic evaluation:* Describe approaches used to estimate resource use associated with the alternative interventions. Describe primary or secondary research methods for valuing each resource item in terms of its unit cost. Describe any adjustments made to approximate to opportunity costs. | | 3-4 |
|  |  | 13b | *Model-based economic evaluation:* Describe approaches and data sources used to estimate resource use associated with model health states. Describe primary or secondary research methods for valuing each resource item in terms of its unit cost. Describe any adjustments made to approximate to opportunity costs. | |  |
| Currency, date, conversion | price and | 14 | Report the dates of the estimated resource quantities and unit costs. Describe methods for adjusting estimated unit costs to the year of reported costs if necessary. Describe methods for converting costs into a common currency base and the exchange rate. | | 3-5 |
| Choice of model | | 15 | Describe and give reasons for the specific type of decision-analytical model used. Providing a figure to show model structure is strongly recommended. | | Figure 1 |
| Assumptions | | 16 | Describe all structural or other assumptions underpinning the decision-analytical model. | | 3-5 |
| Analytical methods | | 17 | Describe all analytical methods supporting the evaluation. This could include methods for dealing with skewed, missing, or censored data; extrapolation methods; methods for pooling data; approaches to validate or make adjustments (such as half cycle corrections) to a model; and methods for handling population heterogeneity and uncertainty. | | 4 |
| **Results** | | | | | |
| Study parameters | | 18 | Report the values, ranges, references, and, if used, probability distributions for all parameters. Report reasons or sources for distributions used to represent uncertainty where appropriate. Providing a table to show the input values is strongly recommended. | | Supplementary Table S7 |
| Incremental costs and outcomes | | 19 | For each intervention, report mean values for the main categories of estimated costs and outcomes of interest, as well as mean differences between the comparator groups. If applicable, report incremental cost-effectiveness ratios. | | Table 2 |
| Characterising uncertainty | | 20a | Single study-based economic evaluation: Describe the effects of sampling uncertainty for the estimated  incremental cost and incremental effectiveness  parameters, together with the impact of  methodological assumptions (such as discount rate,  study perspective). | | Figure 4  6 |
|  | | 20b | *Model-based economic evaluation:* | Describe the |  |
|  | |  | effects on the results of uncertainty for all input parameters, and uncertainty related to the structure of the model and assumptions. | |  |
| Characterising heterogeneity | | 21 | If applicable, report differences in costs, outcomes, or cost-effectiveness that can be explained by variations between subgroups of patients with different baseline characteristics or other observed variability in effects that are not reducible by more information. | | Not applicable |
| **Discussion** | | | | | |
| Study findings,  limitations,  generalisability,  and current  knowledge | | 22 | Summarise key study findings and describe how they support the conclusions reached. Discuss limitations and the generalisability of the findings and how the findings fit with current knowledge. | | 11-12 |
| **Other** | | | | | |
| Source of  funding | | 23 | Describe how the study was funded and the role of the funder in the identification, design, conduct, and reporting of the analysis. Describe other non-monetary sources of support. | | Not applicable |
| Conflicts of  interest | | 24 | Describe any potential for conflict of interest of study contributors in accordance with journal policy. In the absence of a journal policy, we recommend authors comply with International Committee of Medical Journal Editors recommendations. | | Not applicable |

*A good template page for CHEERS Checklist is as follows: <https://www.ispor.org/heor-resources/good-practices/cheers>(2).

**Supplementary Table S5.** Summary of statistical goodness-of-fit of K-M curve.

| Trial | Group | AIC/BIC | Exponential | Gamma | Gen.Gamma | Gompertz | Weibull(PH） | Weibull(AFT） | Log-logistic | Log-normal |
| --- | --- | --- | --- | --- | --- | --- | --- | --- | --- | --- |
| CAPSTONE-1 (OS) | Adebrelimab + Chemotherapy | AIC | 1237.704 | 1207.565 | 1205.509 | 1228.152 | 1212.125 | 1212.125 | 1202.421 | 1203.908 |
|  | Chemotherapy | AIC | 1402.331 | 1320.466 | 1322.466 | 1354.880 | 1325.601 | 1325.601 | 1312.653 | 1335.618 |
|  | Adebrelimab + Chemotherapy | BIC | 1241.140 | 1214.440 | 1215.820 | 1235.030 | 1219.000 | 1219.000 | 1209.300 | 1210.780 |
|  | Chemotherapy | BIC | 1405.780 | 1327.360 | 1332.810 | 1361.770 | 1332.490 | 1332.490 | 1319.550 | 1342.510 |
| KEYNOTE-604 (OS) | Pembrolizumab + Chemotherapy | AIC | 1288.596 | 1281.916 | 1283.771 | 1287.702 | 1282.681 | 1282.681 | 1281.959 | 1294.952 |
|  | Chemotherapy | AIC | 1363.354 | 1324.518 | 1326.375 | 1347.032 | 1328.026 | 1328.026 | 1319.659 | 1341.089 |
|  | Pembrolizumab + Chemotherapy | BIC | 1292.025 | 1288.774 | 1294.059 | 1294.561 | 1289.540 | 1289.540 | 1288.818 | 1301.811 |
|  | Chemotherapy | BIC | 1366.770 | 1331.350 | 1336.620 | 1353.860 | 1334.860 | 1334.860 | 1326.490 | 1347.920 |
| ASTRUM-005 (OS) | Serplulimab + Chemotherapy | AIC | 1240.235 | 1192.898 | 1194.760 | 1206.795 | 1193.390 | 1193.390 | 1191.376 | 1201.594 |
|  | Chemotherapy | AIC | 756.5633 | 727.249 | 729.249 | 738.297 | 728.274 | 728.274 | 726.442 | 732.522 |
|  | Serplulimab + Chemotherapy | BIC | 1244.200 | 1200.830 | 1206.650 | 1214.720 | 1201.320 | 1201.320 | 1199.300 | 1209.520 |
|  | Chemotherapy | BIC | 759.840 | 733.810 | 739.080 | 744.850 | 734.830 | 734.830 | 733.000 | 739.080 |
| RATIONALE-312 (OS) | Tislelizumab + Chemotherapy | AIC | 1382.268 | 1370.993 | 1370.572 | 1382.720 | 1373.745 | 1373.745 | 1363.105 | 1374.189 |
|  | Chemotherapy | AIC | 1498.562 | 1442.852 | 1438.861 | 1478.516 | 1452.074 | 1452.074 | 1435.051 | 1437.650 |
|  | Tislelizumab + Chemotherapy | BIC | 1385.690 | 1377.840 | 1380.850 | 1389.570 | 1380.600 | 1380.600 | 1369.950 | 1381.040 |
|  | Chemotherapy | BIC | 1502.000 | 1449.728 | 1449.175 | 1485.392 | 1458.951 | 1458.951 | 1441.927 | 1444.526 |
| IMpower133 (OS) | Atezolizumab + Chemotherapy | AIC | 827.045 | 807.178 | 805.583 | 805.619 | 804.162 | 804.162 | 807.384 | 832.407 |
|  | Chemotherapy | AIC | 983.693 | 949.354 | 942.520 | 943.484 | 942.139 | 942.139 | 951.387 | 987.237 |
|  | Atezolizumab + Chemotherapy | BIC | 830.350 | 813.780 | 815.490 | 812.230 | 810.770 | 810.770 | 813.990 | 839.010 |
|  | Chemotherapy | BIC | 987.0008 | 955.9705 | 952.445 | 950.100 | 948.756 | 948.756 | 958.004 | 993.853 |
| CASPIAN  (OS) | Durvalumab + Chemotherapy | AIC | 1626.208 | 1613.898 | 1615.785 | 1621.662 | 1614.060 | 1614.060 | 1616.218 | 1641.011 |
|  | Chemotherapy | AIC | 1684.132 | 1649.680 | 1651.612 | 1666.217 | 1650.959 | 1650.959 | 1651.854 | 1676.015 |
|  | Durvalumab + Chemotherapy | BIC | 1629.800 | 1621.080 | 1626.560 | 1628.840 | 1621.240 | 1621.240 | 1623.400 | 1648.190 |
|  | Chemotherapy | BIC | 1687.730 | 1656.870 | 1662.400 | 1673.410 | 1658.150 | 1658.150 | 1659.040 | 1683.200 |

| ETER701  (OS) | Benmelstobart + Anlotinib + Chemotherapy | AIC | 828.608 | 807.130 | 805.231 | 822.028 | 810.161 | 810.161 | 804.789 | 803.233 |
| --- | --- | --- | --- | --- | --- | --- | --- | --- | --- | --- |
|  | Chemotherapy | AIC | 1048.894 | 994.424 | 996.417 | 1016.911 | 996.776 | 996.776 | 991.197 | 1003.785 |
|  | Benmelstobart + Anlotinib + Chemotherapy | BIC | 832.110 | 814.140 | 815.750 | 829.040 | 817.170 | 817.170 | 811.800 | 810.240 |
|  | Chemotherapy | BIC | 1052.400 | 1001.440 | 1006.950 | 1023.930 | 1003.790 | 1003.790 | 998.220 | 1010.800 |
| EXTENTORCH (OS) | Toripalimab + Chemotherapy | AIC | 1400.418 | 1356.327 | 1356.885 | 1386.658 | 1362.716 | 1362.716 | 1345.972 | 1364.214 |
|  | Chemotherapy | AIC | 1444.930 | 1389.871 | 1391.857 | 1418.357 | 1394.179 | 1394.179 | 1383.972 | 1412.276 |
|  | Toripalimab + Chemotherapy | BIC | 1403.830 | 1363.140 | 1367.110 | 1393.470 | 1369.530 | 1369.530 | 1352.790 | 1371.030 |
|  | Chemotherapy | BIC | 1448.320 | 1396.650 | 1402.020 | 1425.130 | 1400.960 | 1400.960 | 1390.750 | 1419.050 |
| CAPSTONE-1 (PFS) | Adebrelimab + Chemotherapy | AIC | 1152.499 | 1129.910 | 1092.740 | 1153.853 | 1142.242 | 1142.242 | 1087.816 | 1096.008 |
|  | Chemotherapy | AIC | 1132.146 | 1013.851 | 1011.708 | 1107.167 | 1040.494 | 1040.494 | 982.045 | 1016.354 |
|  | Adebrelimab + Chemotherapy | BIC | 1155.937 | 1136.786 | 1103.054 | 1160.729 | 1149.119 | 1149.119 | 1094.692 | 1102.885 |
|  | Chemotherapy | BIC | 1135.593 | 1020.744 | 1022.048 | 1114.061 | 1047.388 | 1047.388 | 988.938 | 1023.247 |
| KEYNOTE-604 (PFS) | Pembrolizumab + Chemotherapy | AIC | 1090.623 | 1043.80 | 1045.425 | 1077.063 | 1049.853 | 1049.853 | 1032.178 | 1060.857 |
|  | Chemotherapy | AIC | 1108.967 | 987.157 | 986.944 | 1043.740 | 990.103 | 990.103 | 979.278 | 1021.243 |
|  | Pembrolizumab + Chemotherapy | BIC | 1094.052 | 1050.658 | 1055.713 | 1083.922 | 1056.712 | 1056.712 | 1039.036 | 1067.716 |
|  | Chemotherapy | BIC | 1112.384 | 993.989 | 997.192 | 1050.572 | 996.935 | 996.935 | 986.110 | 1028.075 |
| ASTRUM-005 (PFS) | Serplulimab + Chemotherapy | AIC | 1446.005 | 1360.647 | 1358.640 | 1412.214 | 1372.267 | 1372.267 | 1346.817 | 1360.637 |
|  | Chemotherapy | AIC | 832.124 | 735.557 | 737.483 | 779.438 | 741.639 | 741.639 | 728.708 | 747.846 |
|  | Serplulimab + Chemotherapy | BIC | 1449.969 | 1368.574 | 1370.531 | 1420.141 | 1380.194 | 1380.194 | 1354.744 | 1368.564 |
|  | Chemotherapy | BIC | 835.402 | 742.113 | 747.317 | 785.995 | 748.195 | 748.195 | 735.264 | 754.403 |
| RATIONALE-312 (PFS) | Tislelizumab + Chemotherapy | AIC | 1173.828 | 1173.536 | 1104.707 | 1144.768 | 1175.684 | 1175.684 | 1098.060 | 1112.808 |
|  | Chemotherapy | AIC | 1150.531 | 1031.399 | 987.280 | 1149.499 | 1085.140 | 1085.140 | 941.993 | 987.829 |
|  | Tislelizumab + Chemotherapy | BIC | 1177.253 | 1180.386 | 1114.982 | 1151.618 | 1182.534 | 1182.534 | 1104.910 | 1119.658 |
|  | Chemotherapy | BIC | 1153.969 | 1038.275 | 997.594 | 1156.375 | 1092.016 | 1092.016 | 948.869 | 994.706 |
| IMpower133 (PFS) | Atezolizumab + Chemotherapy | AIC | 979.864 | 932.776 | 932.812 | 971.402 | 942.516 | 942.516 | 911.484 | 944.447 |
|  | Chemotherapy | AIC | 1021.796 | 940.622 | 942.619 | 993.582 | 950.132 | 950.132 | 919.415 | 973.692 |
|  | Atezolizumab + Chemotherapy | BIC | 983.167 | 939.383 | 942.722 | 978.009 | 949.123 | 949.123 | 918.091 | 951.053 |
|  | Chemotherapy | BIC | 1025.104 | 947.238 | 952.543 | 1000.199 | 956.748 | 956.748 | 926.032 | 980.309 |
| CASPIAN  (PFS) | Durvalumab + Chemotherapy | AIC | 1489.873 | 1477.485 | 1444.230 | 1485.796 | 1487.012 | 1487.012 | 1420.025 | 1442.237 |
|  | Chemotherapy | AIC | 1342.748 | 1266.273 | 1261.608 | 1334.519 | 1285.523 | 1285.523 | 1246.720 | 1265.701 |
|  | Durvalumab + Chemotherapy | BIC | 1493.464 | 1484.667 | 1455.003 | 1492.978 | 1494.194 | 1494.194 | 1427.207 | 1449.419 |
|  | Chemotherapy | BIC | 1346.343 | 1273.462 | 1272.392 | 1341.709 | 1292.712 | 1292.712 | 1253.910 | 1272.890 |
| ETER701  (PFS) | Benmelstobart + Anlotinib + Chemotherapy | AIC | 1002.684 | 944.957 | 922.649 | 994.067 | 960.706 | 960.706 | 926.275 | 925.522 |
|  | Chemotherapy | AIC | 1091.6774 | 899.295 | 901.286 | 1029.281 | 926.265 | 926.265 | 871.365 | 918.628 |
|  | Benmelstobart + Anlotinib + Chemotherapy | BIC | 1006.190 | 951.970 | 933.170 | 1001.080 | 967.720 | 967.720 | 933.290 | 932.530 |
|  | Chemotherapy | BIC | 1095.187 | 906.314 | 911.814 | 1036.300 | 933.284 | 933.284 | 878.384 | 925.647 |
| EXTENTORCH (PFS) | Toripalimab + Chemotherapy | AIC | 1303.245 | 1285.917 | 1248.567 | 1302.351 | 1297.318 | 1297.318 | 1234.387 | 1248.627 |
|  | Chemotherapy | AIC | 1217.194 | 1073.486 | 1074.790 | 1133.454 | 1081.053 | 1081.053 | 1070.830 | 1099.951 |
|  | Toripalimab + Chemotherapy | BIC | 1306.652 | 1292.731 | 1258.788 | 1309.166 | 1304.132 | 1304.132 | 1241.201 | 1255.442 |
|  | Chemotherapy | BIC | 1220.583 | 1080.264 | 1084.957 | 1140.233 | 1087.831 | 1087.831 | 1077.609 | 1106.729 |

**Supplementary Table S6.** Optimal Fitting Distributions and Parameters.

| Trial | Group | K-M Curves | Optimal Fitting Distributions | Scale Parameters (λ) | Shape Parameters (γ） |
| --- | --- | --- | --- | --- | --- |
| CAPSTONE-1 | Adebrelimab + Chemotherapy | OS | Log-logistic | 0.063 | 1.956 |
|  | Chemotherapy | OS | Log-logistic | 0.077 | 2.663 |
|  | Adebrelimab + Chemotherapy | PFS | Log-logistic | 0.149 | 2.117 |
|  | Chemotherapy | PFS | Log-logistic | 0.176 | 3.397 |
| KEYNOTE-604 | Pembrolizumab + Chemotherapy | OS | Log-logistic | 0.089 | 1.601 |
|  | Chemotherapy | OS | Log-logistic | 0.101 | 2.143 |
|  | Pembrolizumab + Chemotherapy | PFS | Log-logistic | 0.197 | 2.332 |
|  | Chemotherapy | PFS | Log-logistic | 0.216 | 3.259 |
| ASTRUM-005 | Serplulimab + Chemotherapy | OS | Log-logistic | 0.065 | 2.050 |
|  | Chemotherapy | OS | Log-logistic | 0.087 | 2.088 |
|  | Serplulimab + Chemotherapy | PFS | Log-logistic | 0.153 | 2.346 |
|  | Chemotherapy | PFS | Log-logistic | 0.224 | 3.149 |
| RATIONALE-312 | Tislelizumab + Chemotherapy | OS | Log-logistic | 0.061 | 1.708 |
|  | Chemotherapy | OS | Log-logistic | 0.073 | 2.300 |
|  | Tislelizumab + Chemotherapy | PFS | Log-logistic | 0.175 | 1.843 |
|  | Chemotherapy | PFS | Log-logistic | 0.216 | 3.871 |
| IMpower133 | Atezolizumab + Chemotherapy | OS | Log-logistic | 0.077 | 1.933 |
|  | Chemotherapy | OS | Log-logistic | 0.096 | 2.192 |
|  | Atezolizumab + Chemotherapy | PFS | Log-logistic | 0.190 | 2.523 |
|  | Chemotherapy | PFS | Log-logistic | 0.213 | 3.030 |
| CASPIAN | Durvalumab + Chemotherapy | OS | Log-logistic | 0.079 | 1.690 |
|  | Chemotherapy | OS | Log-logistic | 0.095 | 2.005 |
|  | Durvalumab + Chemotherapy | PFS | Log-logistic | 0.172 | 1.978 |
|  | Chemotherapy | PFS | Log-logistic | 0.190 | 2.584 |
| ETER701 | Benmelstobart + Anlotinib + Chemotherapy | OS | Log-logistic | 0.057 | 1.849 |
|  | Chemotherapy | OS | Log-logistic | 0.082 | 2.354 |
|  | Benmelstobart + Anlotinib + Chemotherapy | PFS | Log-logistic | 0.133 | 2.447 |
|  | Chemotherapy | PFS | Log-logistic | 0.220 | 4.232 |
| EXTENTORCH | Toripalimab + Chemotherapy | OS | Log-logistic | 0.066 | 2.220 |
|  | Chemotherapy | OS | Log-logistic | 0.076 | 2.367 |
|  | Toripalimab + Chemotherapy | PFS | Log-logistic | 0.158 | 2.093 |
|  | Chemotherapy | PFS | Log-logistic | 0.185 | 3.403 |

**Supplementary Table S7.** Model Parameters: Clinical and Cost data.

| Parameters | Baseline Value | Lower Limits | Upper Limits | Distribution | Parametric sources |
| --- | --- | --- | --- | --- | --- |
| Costs (US) | | | | | |
| Adebrelimab (12ml:600mg) | 1313.97 | 1051.18 | 1576.76 | Gamma | Local Price(3) |
| Pembrolizumab (4ml:100mg) | 2478.28 | 1982.63 | 2973.94 | Gamma | Local Price(3) |
| Serplulimab (10ml:100mg) | 772.89 | 618.31 | 927.47 | Gamma | Local Price(3) |
| Tislelizumab (10ml:100mg) | 173.38 | 138.70 | 208.05 | Gamma | Local Price(3) |
| Atezolizumab (20ml:1.2g) | 4536.65 | 3629.32 | 5443.98 | Gamma | Local Price(3) |
| Durvalumab (10ml:500mg) | 2501.69 | 2001.35 | 3002.02 | Gamma | Local Price(3) |
| Benmelstobart (20ml:600mg) | 1698.48 | 1358.78 | 2038.17 | Gamma | Local Price(3) |
| Anlotinib (12mg,7 tablets per box) | 274.48 | 219.59 | 329.38 | Gamma | Local Price(3) |
| Toripalimab (6ml:240mg) | 260.70 | 208.56 | 312.84 | Gamma | Local Price(3) |
| Etoposide (5ml:100m) | 1.08 | 0.86 | 1.29 | Gamma | Local Price(3) |
| Carboplatin (50mg) | 5.81 | 4.65 | 6.97 | Gamma | Local Price(3) |
| Cisplatin (10mg) | 1.10 | 0.88 | 1.32 | Gamma | Local Price(3) |
| Topotecan (2mg) | 14.20 | 11.36 | 17.03 | Gamma | Local price(3) |
| Follow-up testing cost per 3-week cycle (US)^a^ | 55.60 | 44.48 | 66.72 | Gamma | Luo X  *et al*(4) |
| Subsequent therapy cost per 3-week cycle (US)^b^ | 854.05 | 683.24 | 1024.86 | Gamma | Luo X  *et al*(4) |
| BSC cost per 3-week cycle (US)^c^ | 337.50 | 270.00 | 405.00 | Gamma | Luo X  *et al*(4) |
| Treatment costs of adverse events during treatment (US)^d^ | 2794.00 | 2235.20 | 3352.80 | Gamma | Wong W *et al* (5) |
| Clinical data | | | | | |
| Utility of PFS | 0.70 | 0.56 | 0.84 | Beta | Vedadi A *et al* (6) |
| Utility of PD | 0.60 | 0.48 | 0.72 | Beta | Vedadi A *et al* (6) |
| Discount rate | 0.05 | 0.04 | 0.06 | Beta | China Guidelines for Pharmacoeconomic Evaluations  (7) |
| Creatinine clearance rate (ml/min) | 80.00 | 64.00 | 96.00 | Normal | Anderton JL *et al* (8) |
| Body surface area (m**^2^**) | 1.72 | 1.38 | 2.06 | Normal | The State Council Information Office of the People’s Republic of China (9) |
| Mean weight (kg) | 64.00 | 51.20 | 76.80 | Normal | The State Council Information Office of the People’s Republic of China (9) |

^a^ The cost of follow-up testing included outpatient physician visits, laboratory tests and examinations.

^b^ The cost of subsequent therapy referred to additional treatments, such as radiotherapy.

^c^ The cost of BSC referred to the intervention of cancer-related symptoms, including anti-inflammatory treatment, analgesic treatment, antiemetic treatment, thoracic or abdominal paracentesis, blood transfusion and nutritional support, and others.

^d^ The treatment cost of adverse events during treatment referred to the aggregated cost of common adverse events observed in either the experimental or control groups.

PFS, progression-free survival; PD, progressive disease; BSC, best supportive care.

Reference:

1. The PRISMA extension statement for reporting of systematic reviews incorporating network meta-analyses of health care interventions: checklist and explanations - PubMed [Internet]. [cited 2025 Sept 25]. Available from: <https://pubmed.ncbi.nlm.nih.gov/26030634/>

2. Consolidated health economic evaluation reporting standards (CHEERS)--explanation and elaboration: a report of the ISPOR health economic evaluation publication guidelines good reporting practices task force - PubMed [Internet]. [cited 2025 Sept 25]. Available from: <https://pubmed.ncbi.nlm.nih.gov/23538175/>

3. MoShang Pharma (formerly Yaorongyun) - A Full Industrial Chain Data Service Platform for Biomedicine MoShang Pharma Biomedical Database [Internet]. [cited 2025 Sept 25]. Available from <https://www.pharnexcloud.com>.

4. Luo X, Liu Q, Zhou Z, Yi L, Peng L, Wan X, et al. Cost-effectiveness of bevacizumab biosimilar LY01008 combined with chemotherapy as first-line treatment for Chinese patients with advanced or recurrent nonsquamous non-small cell lung cancer. Front Pharmacol. 2022 Apr 19;13:832215.

5. Wong W, Yim YM, Kim A, Cloutier M, Gauthier-Loiselle M, Gagnon-Sanschagrin P, et al. Assessment of costs associated with adverse events in patients with cancer. De Mello RA, editor. PLOS One. 2018 Apr 13;13(4):e0196007.

6. Vedadi A, Shakik S, Brown MC, Lok BH, Shepherd FA, Leighl NB, et al. The impact of symptoms and comorbidity on health utility scores and health-related quality of life in small cell lung cancer using real world data. Qual Life Res Int J Qual Life Asp Treat Care Rehabil. 2021 Feb;30(2):445–54.

7. Chinese Pharmaceutical Association (CPA). China Guidelines for Pharmacoeconomic Evaluations (2020). Beijing.

8. Anderton JL, Gill M, Notghi A. Renal haemodynamic effects of bunazosin retard and prazosin in mild to moderately hypertensive patients with normal or moderately impaired renal function. Nephrol Dial Transplant Off Publ Eur Dial Transpl Assoc - Eur Ren Assoc. 1994;9(6):607–12.

9. The State Council Information Office of the People’s Republic of China. Press conference on the Report on Chinese Residents’ Chronic Diseases and Nutrition 2020. https://www.gov.cn/ [Accessed Dec 23,2020]
